# Simultaneously enhanced tenacity, rupture work, and thermal conductivity of carbon nanotube fibers by raising effective tube portion

**DOI:** 10.1126/sciadv.abq3515

**Published:** 2022-12-14

**Authors:** Xiao Zhang, Michael De Volder, Wenbin Zhou, Liron Issman, Xiaojun Wei, Adarsh Kaniyoor, Jeronimo Terrones Portas, Fiona Smail, Zibo Wang, Yanchun Wang, Huaping Liu, Weiya Zhou, James Elliott, Sishen Xie, Adam Boies

**Affiliations:** ^1^ Beijing National Laboratory for Condensed Matter Physics, Institute of Physics, Chinese Academy of Sciences, Beijing 100190, China.; ^2^ Department of Engineering, University of Cambridge, Cambridge CB2 1PZ, UK.; ^3^ MOE Key Laboratory of Enhanced Heat Transfer and Energy Conservation, Beijing Key Laboratory of Heat Transfer and Energy Conversion, Beijing University of Technology, Beijing 100124, China.; ^4^ Department of Materials Science and Metallurgy, University of Cambridge, Cambridge CB3 0FS, UK.

## Abstract

Although individual carbon nanotubes (CNTs) are superior to polymer chains, the mechanical and thermal properties of CNT fibers (CNTFs) remain inferior to synthetic fibers because of the failure of embedding CNTs effectively in superstructures. Conventional techniques resulted in a mild improvement of target properties while degrading others. Here, a double-drawing technique is developed to rearrange the constituent CNTs. Consequently, the mechanical and thermal properties of the resulting CNTFs can simultaneously reach their highest performances with specific strength ~3.30 N tex^−1^ (4.60 GPa), work of rupture ~70 J g^−1^, and thermal conductivity ~354 W m^−1^ K^−1^ despite starting from low-crystallinity materials (*I*_G_:*I*_D_ ~ 5). The processed CNTFs are more versatile than comparable carbon fiber, Zylon and Dyneema. On the basis of evidence of load transfer efficiency on individual CNTs measured with in situ stretching Raman, we find that the main contributors to property enhancements are the increasing of the effective tube contribution.

## Teaser

With Double-Drawing, carbon nanotube fibers become competitive and simultaneously strong, tough, and thermally conductive.

## INTRODUCTION

Carbon nanotube (CNT) macroscopic assemblies, like CNT fibers (CNTFs), are analogous to bulk materials of highly conjugated polymer molecules (*1*). Correspondingly, constituent CNTs are akin to polymer chains but with outstanding mechanical (*2*–*4*), thermal (*5*), and electrical properties (*6*) and chemical resilience (*7*). Although direct-spun CNTFs can be produced continuously (1 to 2 km hour^−1^) with low cost (*8*–*10*), they still suffer from a one– to two–order of magnitude degradation in properties relative to CNT bundles [tensile strength, 27 to 31 N tex^−1^ and Young’s modulus, 337 to 640 N tex^−1^ (*11*, *12*)] and are uncompetitive with commercial carbon fibers (CFs), Kevlar fibers, and Zylon fibers on their corresponding specialties.

Researchers attribute the property degradation primarily to the poor arrangement of the constituent CNTs within the as-synthesized CNTFs (*1*), and thus, they used various post-synthesis treatments seeking to rearrange the individual CNTs (iCNTs) and CNT bundles. However, with techniques commonly used on textile fibers [e.g., direct stretching (*13*, *14*), compression (*15*–*17*), and twisting (*18*, *19*)], only minor enhancements have been achieved. In 2013, with a similar method to produce Kevlar and Zylon, the solution-spinning technique was reported to produce highly aligned and compacted CNTFs (*20*) from a liquid crystal solution of CNTs in chlorosulfonic acid (CSA). The reported mechanical properties increased substantially to strengths ~1.0 GPa (tenacity ~0.97 N tex^−1^) and moduli ~120 GPa, which heavily rely on the perfect crystalline CNT raw materials used. Recently, researchers stretched the direct-spun CNTFs in CSA to obtain an aligned structure (*21*), which used screening of van der Waals (vdW) forces in CSA (*22*, *23*), similar to stiffening cellulose fibers within water to screen hydrogen bonds between chains (*24*). Although the reported mechanical performances surpass those of solution-spinning CNTFs, superior performance seems only achievable on very thin CNTFs [linear density (*LD*) ~ 0.05 tex], while those of thick CNTFs are much degraded (*25*). However, with higher production rates and efficiencies, macroscale CNTFs with poorer crystalline CNTs (*I*_G_:*I*_D_ < 10) and greater *LD*s (>0.3 tex) deserve to be drastically improved on properties to meet a wide range of applications. In previous reports, besides the limited density achieved, CSA appears to have competing effects, facilitating rearrangement while hindering the load transfer within CNTFs in internal regions where remnant CSA screens vdW forces (*26*). In addition, because phonon conduction is also impeded by the disordered microstructure in CNTFs, strategies to enhance thermal conductivity also warrant investigation. Thus, improving the properties of CNTFs while avoiding the disturbance from residual CSA is the primary motivation of our work.

In addition, while many improvements have been reported for CNT performance, a mechanistic explanation of property enhancement is still incomplete. Although performance improvements were commonly explained by the alignment and compactness increase, most conceptual models omit the distinctive structure of CNTFs as a fibrillar network of crumpled long rigid iCNTs, which is distinct from CF, Kevlar, or cotton yarn. The long iCNTs can span across different bundles and thus behave differently along segments. Furthermore, the efficiency changes of load transfer between iCNTs before and after enhancement and focus on the different behavior of iCNTs under various loads remain to be fully investigated. Thus, our work also focuses on optimizing the mechanism applicable to hierarchical CNT networks bonded by vdW forces to guide further enhancement and to assess current processing techniques.

Here, we develop a novel double-drawing technique to rearrange the iCNTs within raw direct-spun CNTFs, seeking a simultaneous improvement in mechanical (strength, modulus, and toughness) and thermal properties. The iCNT alignment and CNTF porosity were monitored by wide-angle X-ray diffraction (WAXD), small-angle Xray scattering (SAXS) and focused ion beam (FIB) cross-section analysis, as well as in situ stretching Raman (ISSR) to study quantitatively the iCNTs’ behavior in various CNTFs after different levels of processing and loading. Last, on the basis of the experimental findings, we complement the mechanism of enhancement with two critical factors: the increased proportion of load-bearing CNT bundles and the extension of effective length of tubes attached on these bundles.

## RESULTS

### Enhancement of CNTF with the double-drawing process

**Fig. 1.**
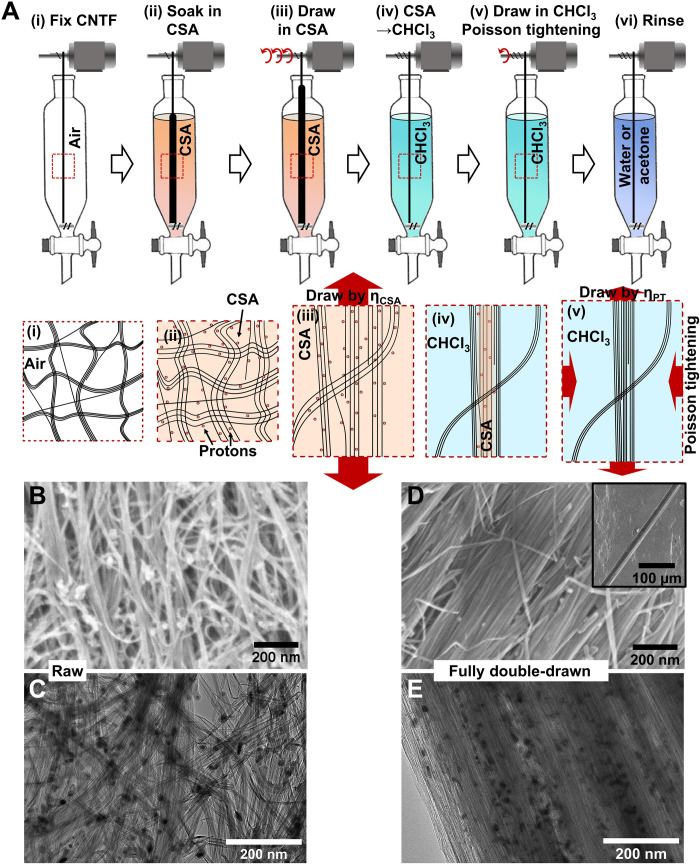
The double-drawing process on CNTF. (**A**) Schematic diagram of the double-drawing process to enhance CNTFs and the corresponding typical cell within CNTF (second row): (i) The raw CNTF is fixed within a dropping funnel straightened out but without a pretension; (ii) to weaken the binding between iCNTs, the raw CNTF is immersed in HSO_3_Cl (CSA); (iii) CNTF is then first drawn in CSA to a specific ratio (*η*_CSA_) to straighten out and align the crumpled iCNTs; (iv) after the immersing solvent is changed into CHCl_3_ (chloroform), (v) CNTF is further drawn by *η*_PT_ = 0.5%, referred to as Poisson tightening. During this process, the remaining CSA is removed from the fiber, which radially compresses and “freezes” iCNTs into larger bundles; (vi) last, CNTF is successively rinsed in water and acetone and vacuum-dried. The substantial enhancement on alignment and compactness of fully double-drawn CNTF (DD-CNTF) can be observed on the surface and inner microstructures by (**B** versus **D**) SEM and (**C** versus **E**) HRTEM. The red arrows in the schematic cell (the second row) represent the force exerted.

In our work, the raw CNTFs are fabricated by direct spinning CNT aerogels produced using a continuous floating catalyst chemical vapor deposition (FCCVD) method. The FCCVD method is considered highly suitable for the continuous mass production of iCNTs with a very high aspect ratio (*1*). These grown CNTs aggregate into bundles and then entangle as an aerogel (*27*, *28*), which is subsequently densified into fibers by acetone during collection from the reactor. The as-synthesized CNTFs consist of a hierarchical network of curled randomly connected CNTs [Fig. 1, A (i), B, and C]. Analogous to polymer molecules in a textile fiber being held together by hydrogen bonds and/or vdW forces, the iCNTs in CNTF are held together by vdW forces in an entangled network. The vdW forces “freeze” CNTF in a nonequilibrium curved morphology, i.e., an athermal structure (*29*).

Here, to enhance CNTF after synthesis, the connections or constraint intra- and inter-bundles are first weakened or even released by immersing CNTF in HSO_3_Cl (CSA) (Fig. 1A, ii). The protonation of CNTs by CSA (*22*) screens the vdW forces, as observed by the swelling of CNTF in CSA. The weakened tube-tube forces thus reduce the large shear strength. Thus, the mutual lateral movement of iCNTs can occur easily, which otherwise might break CNT bundles if done without CSA. This process is analogous to the high-humidity environment softening the hydrogen bonds between cellulose molecules before enhancing cellulose fiber.

CNTF is then first drawn in CSA to a specific draw ratio (η_CSA_, the extension length divided by original length), during which the crumpled CNTs are freely straightened and aligned along the fiber axis (Fig. 1A, iii), with minimal breaking of CNT bundles. To quantitatively study the effect of drawing in CSA, the range of η_CSA_ is controlled from 0% to the maximum ratio η_max_, in which η_max_ is ~3% less than the failure ratio in CSA (η*). We find a strong dependence of η* on *LD* of CNTF (details can be found in text S1). For CNTFs, the respective dependencies on *LD* and draw ratios are observed *LD* ~ 0.5 tex, η* ~ 28%, and η_max_ ~ 25%.

After drawing, the remaining CSA within CNTF still hinders the load transfer between iCNTs caused by vdW forces. Chloroform serves as the best solvent to dissolve CSA, but the fine voids in the increasingly compacted outer CNT layers hinder the outward diffusion of CSA (Fig. 1A, iv). Therefore, to remove the remaining CSA and densify the fiber, we further introduce a second drawing process, referred to as the “Poisson tightening” process. After the first drawing in CSA, the fiber is immediately drawn further in chloroform with another draw ratio, η_PT_ ~ 0.5% (Fig. 1A, v). When immersed in chloroform, vdW forces among CNTs in the outer layer start to recover, thus increasing the layer’s modulus. Then, under subsequent axial drawing, the resulting radial tightening, caused by the Poisson effect, further expels CSA while stiffening and compressing CNTs in the inner layers (details in text S1). We also find that excessive drawing, η_PT_ > 0.5%, may cause plastic deformation and break CNT bundles. After the above double-drawing processes, CNTFs are successively rinsed with water and acetone (Fig. 1A, vi) and finally vacuum-dried for further use.

### The microstructure after drawing

**Fig. 2.**
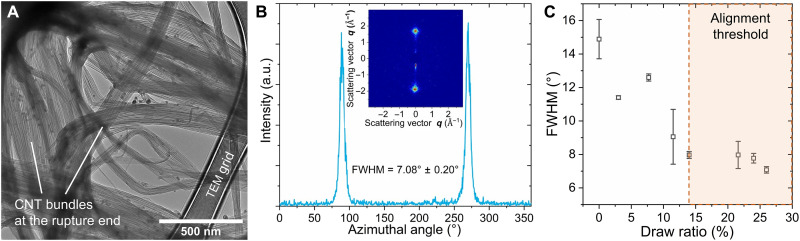
Microstructure of CNTF after the double-drawing process. (**A**) The fracture end of the fully DD-CNTF illustrates fringed morphology with thick bundles commonly seen in the highly oriented linear polymer fibers. Within every large bundle, the iCNTs closely compact into thick bundles in a range of 50 to 200 nm, much larger than those of raw CNTFs. (**B**) The wide-angle X-ray diffraction (WAXD) patterns (inset) of the suspended CNTFs illustrate the two preferred orientation peaks of the (002) planes in the azimuthal profile. The full width at half maximum (FWHM) of the peaks indicates the level of alignment within CNTFs. (**C**) With the gradual increase in the draw ratio, the alignment of iCNTs increases but reaches a plateau state after threshold level of drawing (~14.5%).

For the double-drawn CNTF (DD-CNTF) with full drawing (η_CSA_ = η_max_ = 25% and η_PT_ = 0.5%), referred to as fully DD-CNTF, both the mesoscale and nanoscale structures are organized. The disordered network of the raw CNTF is optimized into an aligned and tightly compacted bundle structure (Fig. 1, B to E). The raw thin bundles (diameter of 10 to 50 nm; Fig. 1C) converge to much thicker bundles with a diameter of 80 to 500 nm, as observed in the fracture end of the fully DD-CNTF (Fig. 2A). Within the thick bundles, all iCNTs are tightly packed.

The alignment evolution within CNTFs is observed by wide-angle x-ray diffraction (WAXD) (*13*). The full width at half maximum (FWHM) of the preference peak in azimuthal scan is an indicator of CNT alignment (Fig. 2B). As the draw ratio increases to 14.5%, FWHM decreases from 14.89° to 7.97° (Fig. 2C). At draw ratios above 14.5%, FWHM plateaus near 7.9°, which indicates the saturation of the alignment of CNTs above the threshold level of drawing. Similar results are shown for SAXS (fig. S1), where the optimization of alignment levels off above 12% of drawing.

**Fig. 3.**
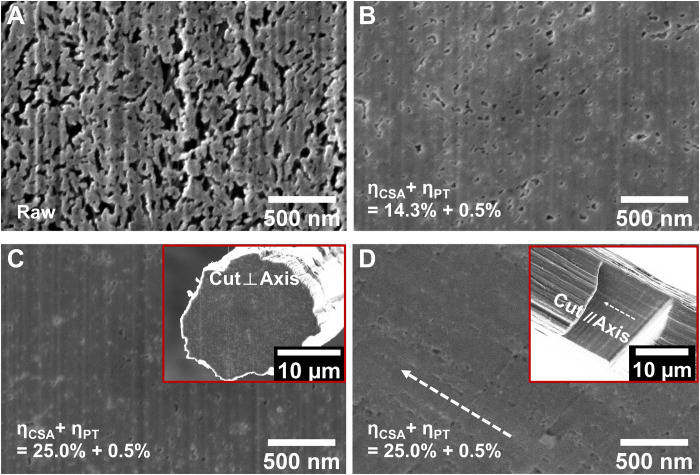
The evolution of porosity within CNTF after various levels of double drawing. (**A** to **C**) On the cross-section cut perpendicular to the fiber axis, the porosity monotonically decreases with the increase of draw ratio. (C) For the fully DD-CNTF, only fine voids can be found. (**D**) On the cross-section cut parallel to the fiber axis as shown in the inset, the remaining voids are all in a configuration of bead chain along the fiber axis (white arrow) instead of randomly distributed, indicating their presence between large bundles and aerogel layers.

The evolution of voids within DD-CNTF was monitored by scanning electron microscopy (SEM) of the cross-section cut by FIB. As shown in Fig. 3 (A to C), with increasing draw ratio, the porosity decreases and reaches the minimum on the fully DD-CNTF with fine voids (more details can be found in fig. S2). Because the area surrounded by the voids is an indicator of the cross section of a bundle, the ever-increasing solid area also illustrates the thickening of bundles. We further checked the cross-section cut parallel to the fiber axis (Fig. 3D). The remaining voids are in bead-chain configuration, indicating that the remaining voids in the fully DD-CNTF originate from the gaps between thick bundles and layers of aerogel.

### Enhancement of mechanical properties for DD-CNTFs

The strength of fibers increases with the increasing density where the cross-sectional area diminishes for a given fiber as it is densified (*30*). The cross-sectional area is poorly defined for porous nanomaterials and assemblies with ambiguous cross-sections or nonuniform diameters. Thus, to best characterize DD-CNTFs, “tenacity,” i.e., “specific strength” with units of N tex^−1^ (or GPa SG^−1^, where SG is the specific gravity relative to water), is a well-defined indicator of load and does not have the ambiguity of absolute “strength” with units of GPa. Tenacity is widely used for textile fibers and can be calculated directly by dividing stretching force with *LD* (mass/length), both of which can be unambiguously measured for fibers and porous nanomaterials.

**Fig. 4.**
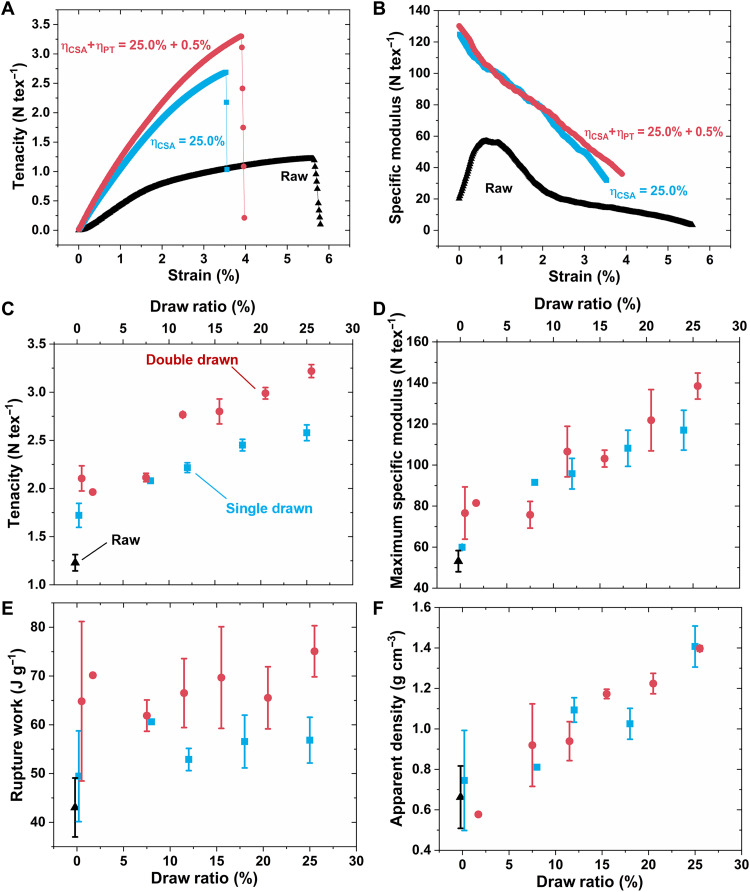
The evolution of the mechanical properties of CNTF with various draw ratios. (**A**) In the representative tenacity-strain (ε) plots of CNTFs, for DD-CNTFs (red dots) and SD-CNTFs (blue squares), they are both notablely stiffened compared with the raw fiber (black triangles). With additional Poisson tightening, the breaking tenacity further improved by 23% without sacrificing ductility. (**B**) In the corresponding tangent-specific modulus-strain plots, after drawing in CSA, the tangent-specific modulus dropped monotonically with the increase of strain. (**C**) With CNTFs after different drawings, the evolution of the breaking tenacity and (**D**) the initial specific modulus versus the draw ratios (η_CSA_ + η_PT_) are shown. Both DD-CNTF and SD-CNTF tenacity and the initial modulus monotonically increase with the increasing draw ratio. (**E**) The work of rupture after Poisson tightening increases, particularly for CNTF above threshold drawing. (**F**) With the organized microstructure, the apparent density increases monotonically with the draw ratio and reaches 1.40 g cm^−3^ for the fully DD-CNTF.

As shown in Fig. 4A, compared with the raw CNTFs (black triangles), after full double drawing (red dots), the fibers exhibit an increase in breaking tenacity from 1.22 to 3.30 N tex^−1^. When compared with CNTF only drawn in CSA [single-drawn CNTF (SD-CNTF); blue squares in Fig. 4], the tenacity of the fully DD-CNTF has 23% greater tenacity without any ductility degradation, which illustrates the importance of the Poisson tightening process. Fiber strength, when accounting for SEM-measured cross-sectional areas, increases from raw fiber strength of 0.9 to 4.6 GPa for fully DD-CNTFs (see fig. S3).

With the gradual increase in the draw ratio, both DD-CNTFs and SD-CNTFs show a monotonic increase in tenacity (Fig. 4C). We find no saturation plateau on tenacity after threshold drawing, as was shown with alignment (Fig. 2C). The further enhancing effect from Poisson tightening is more obvious when η_CSA_ > 10%. Furthermore, because of the consistency of ductility before and after the Poisson tightening (fig. S4), the work of rupture, i.e., energy absorbed during the rupture process, increased to ~70 J g^−1^ for DD-CNTFs, from 42 J g^−1^ for the raw CNTF, and ~55 J g^−1^ for SD-CNTFs.

In the corresponding tangent-specific modulus-strain plots (Fig. 4B) for both DD-CNTFs and SD-CNTFs, the modulus reduces during the tensile testing process. The rising of modulus for the raw CNTF at the beginning comes from the deformation of the iCNT network (*31*). For CNTFs after different draw ratios, the initial modulus (maximum modulus) monotonically increases with the rising of draw ratio (Fig. 4D) and reaches 130.2 N tex^−1^ for the specific modulus of fully DD-CNTF, compared with 56.8 N tex^−1^ for the raw CNTF. The close stacking and collapsed cross section (fig. S5) increase the CNTF apparent density from 0.66 g cm^−3^ of the raw fibers to 1.40 g cm^−3^ of the fully DD-CNTF (Fig. 4F), which is much closer to the theoretical density of multiwalled CNTs (*32*) (~1.7 g cm^−3^ for our multiwalled CNTs; more information in fig. S11) than recent reports (*23*,*28*).

It is also interesting that by only immersing in CSA, CNTF gains ~40% increase in tenacity (Fig. 4C), along with short and straight CNT bundles on the fiber surface (Fig. 1D). We believe that these phenomena originate from the spontaneous rearrangement of iCNTs due to their high stiffness and persistence length (more discussion in text S3) (*29*, *33*).

### Enhancement of thermal properties for DD-CNTFs

**Fig. 5.**
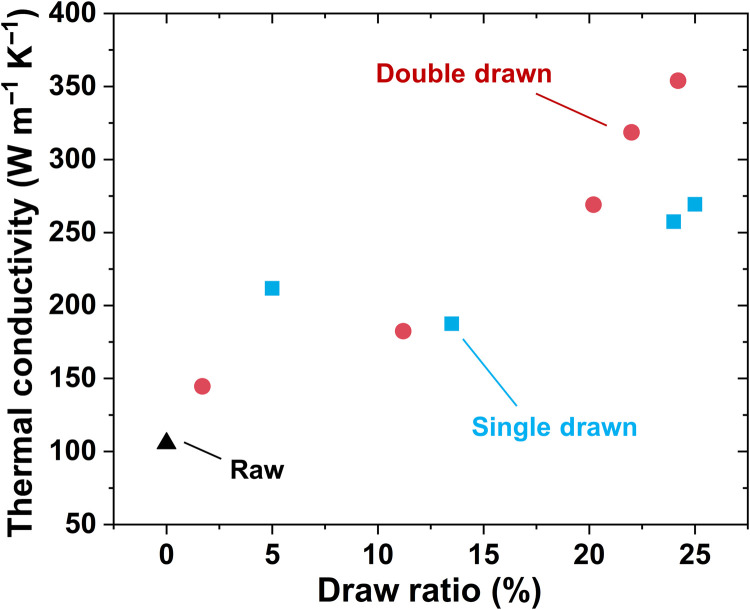
Evolution of thermal conductivity with the increase of draw ratio. Owing to the optimization of CNT-CNT junctions after drawing, the thermal conductivity of the fully DD-CNTF reaches 353.9 W m^−1^ K^−1^, which is 335% higher than the raw CNTF and 31% higher than the fully SD-CNTFs.

Since the CNT-CNT contacts (junctions) are the main source of thermal resistance (*34*) [e.g., phonon scattering centers (*35*)], the fiber thermal conductivity also improves from the microstructure rearrangement after drawing. With the gradual increase of draw ratio, the thermal conductivity increases monotonically, and the Poisson tightening gives rise to further increase after the threshold drawing (Fig. 5). Consequently, thermal conductivity of the fully DD-CNTF reaches 354 W m^−1^ K^−1^, which is 335% higher than the raw CNTF and 31% higher than the fully SD-CNTFs. Normalized by density, the specific thermal conductivity of the fully DD-CNTF (0.258 W m^2^ K^−1^ kg^−1^) is five times higher than that of copper (0.044 W m^2^ K^−1^ kg^−1^) and silver (0.041 W m^2^ K^−1^ kg^−1^). The electrical conductivity also substantially increases from 1650 S cm^−1^ of the raw CNTF to 10,700 S cm^−1^ of the fully DD-CNTF (fig. S6). While improved, the electrical conductivity remains below the conductivity of other bulk metal conductors, e.g., copper (~600,000 S cm^−1^). In contrast to alignment evolution with the draw ratio, the evolution of mechanical and conductive properties monotonically improves, which implies that there are other enhancing mechanisms besides the improvement in alignment.

### Comparison between the DD-CNTF with commercial fibers

**Fig. 6.**
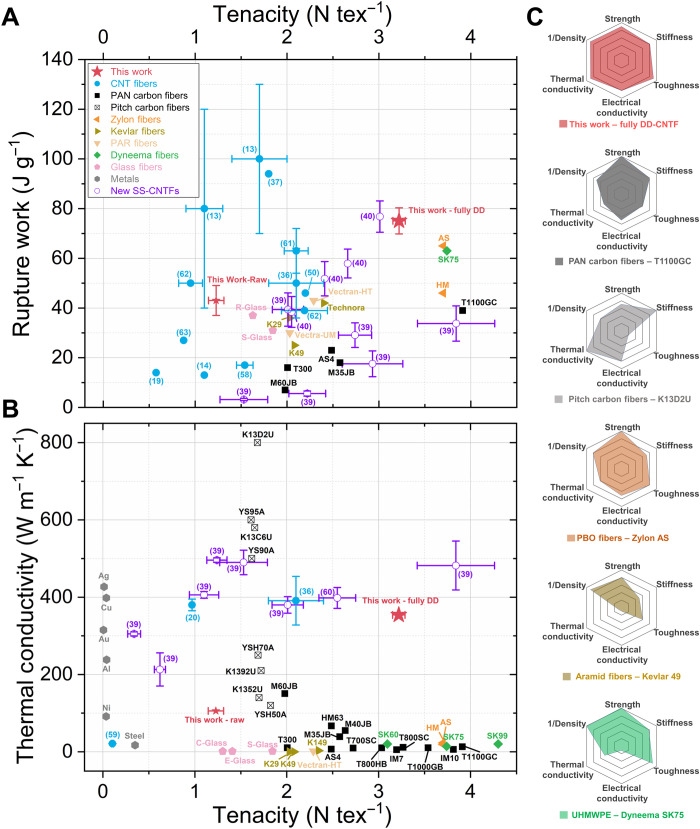
Property comparison between CNTFs and commercial synthetic fibers. Ashby plots of (**A**) tenacity (specific tensile strength) versus work of rupture, and (**B**) tenacity versus thermal conductivity. The data points contain reported high-performance CNTFs (pure), high-strength CFs (PAN-based CFs), highly thermal conductive CFs (pitch-based CFs), some high-performance polymer fibers, and results from this work. (**C**) Radar plots comparing the performance of the fully DD-CNTF with PAN-based CF (T1100GC), pitch-based CF (K13D2U), Zylon AS, Kevlar 49, and Dyneema SK75. The results are normalized by the maximum value of each characteristic (references in table S1).

As shown in Fig. 6, compared with current leading fibers, CNTFs can exhibit overall versatile performance with a combination of high specific tensile strength (tenacity), work of rupture, thermal conductivity, and specific volume (e.g., low density). The fully DD-CNTFs have thermal conductivities that match the best pitch-based CFs and surpass them in terms of tenacity and ductility. The fully DD-CNTF tenacity is within 15% of the strongest polyacrylonitrile (PAN)-based CFs (T1000GB) and polybenzoxazole (PBO) fibers (Zylon AS and HM), but tougher and more conductive. The performance of fully DD-CNTFs is superior to that of Kevlar for all reported metrics, highlighting potential applications of impact shielding or advanced structural usage with thermal management purposes.

The DD-CNTFs further the trend of the remarkable annual improvements of CNTF properties highlighted by Taylor *et al.* (*36*) over the past decade. DD-CNTF is a method that enhances commercially produced raw fibers with fiber test lengths (10 to 20 mm) from medium-grade crystalline materials (*I*_G_:*I*_D_ ~ 5.3; fig. S7). When compared to congeneric fibers, including the solution-spun CNTF composed of higher crystallinity CNTs (*I*_G_:*I*_D_ = 54 to 85) (*36*), the fully DD-CNTFs have improved strength and modulus, owing to the longer effective length of CNTs. Compared to other CNTF acid stretching processes, the DD-CNTF enables a larger *LD* while improving the overall fiber properties. Short CNT strands (VA-MIT) have higher work of rupture than DD-CNTFs (94 J g^−1^) but compromise strength and modulus with short fibers that were restricted lengths less than the iCNTs (~1 mm) (*37*). Critically, the present work enables sustained strength over gauge lengths that exceed the longest CNTs and offer a measure of scaled fiber performance that is comparable (0 to 25% less) to high work of rupture of the IMDEA materials. Our materials do not require removal of catalyst or other impurities from CNTFs, which account for 7 to 8 weight % with marginally crystalline material.

Moreover, we find it worthy to notice the importance of accurate measurement of *LD* because of the susceptibility of fiber’s tenacity to *LD*. In our measurement, the *LD* of every fiber was measured by the direct single-fiber weighing method following ASTM D1577-07(2018) OPTION B using an ultramicrobalance with accuracy down to 0.1 μg. The frequently used vibroscopic method in reports is abandoned here because of the potential serious underestimation of *LD*, if the “stiffness correction” was overlooked [ASTM D1577-07(2018) OPTION C—the standard for the vibroscopic method]. The underestimated *LD* leads to the overestimation of tenacity. Because the vibroscopic method is built on the basis of perfectly flexible string model, measurement deviation can be serious with one of following conditions: ① specimen with high Young’s modulus, ② tested with relative short gauge length, and ③ tested with low pretension. Unfortunately, the enhanced CNTFs always involve at least one of them (more discussion can be found in text S2 and table S2).

If we only focus on the competition between numerical values of reported results, our DD-CNTFs’ performance matches the best results reported but is achieved on the basis of the mass-produced fibers with poor crystallinity (*I*_G_:*I*_D_ ~ 5) and much larger *LD* ~ 0.42 tex, enabling potential for further mass production and real applications. As shown in table S2, it can be easily concluded that with similar *LD* ~ 0.5 tex, the enhanced tenacity is always below 1.6 N tex^−1^ compared with our 3.3 N tex^−1^. With similar crystallinity (*I*_G_:*I*_D_ ~ 5) to ours, the enhanced tenacity also dropped seriously even on thin CNTFs.

During the reviewing process of our work, three new articles focusing on solution-spun (SS)-CNTFs were published. In one article, a new versatile and environment-friendly acid solvent was presented to replace the troublesome CSA solvent (*38*), which will definitely simplify the procedures and facility requirement when enhancing CNTFs, including ours. Another article coalesces iCNTs using 1400° to 2700°C heating, enhancing the shear strength between iCNTs, substantially increasing the modulus of SS-CNTFs (*39*). On SS-CNTFs with *LD* ~ 0.19 tex with heating to 1700°C, they achieved high tenacity (3.84 N tex^−1^) and thermal conductivities (482 W m^−1^ K^−1^), although the ductility and work of rupture are partially sacrificed. The last article used the CNT and graphene oxide hybrid liquid crystal solution to produce hybrid fibers (*40*). They believe that the flexible graphene oxides intercalate between nanotubes, which maximizes the contact between elements. With the optimized graphene oxide content (10 volume %), high tenacity (3.01 N tex^−1^, in table S1 in the Supplemental Materials) and rupture work (76.8 ± 6.3 J g^−1^) can be achieved. All the results newly reported have been added into Fig. 6 (colored in purple).

### Load transfer efficiency on individual tubes as illustrated by the ISSR

The distinctive increase in mechanical properties after the double-drawing process warrants a mechanistic study of the enhancing mechanisms to determine whether the DD-CNTFs have taken the full advantage of iCNTs’ properties. Here, we use the ISSR with the polarized detecting configuration to study the load transfer efficiency on iCNTs. In contrast to the common polarized Raman characterization that solely depicts the CNT alignment in fibers, ISSR enables assessment of the distribution of strain on iCNTs (*41*). In ISSR, the C–C bonds soften under stretching, redshifting the Raman *G*′ mode proportionally with strain (*42*).

**Fig. 7.**
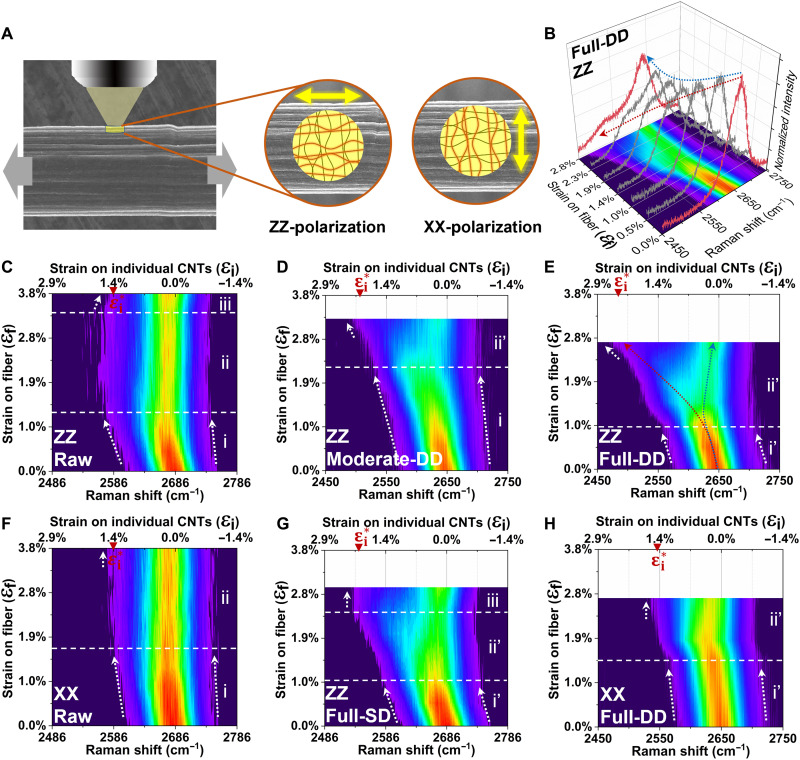
The redshift of Raman *G*′ mode of CNTFs evaluated by the ISSR. (**A**) Schematic diagram of the ISSR characterization with polarized laser and ZZ/XX configuration. (**B**) For the fully DD-CNTF, with the increase of strain on CNTF (ε_f_), the redshift is distinctively efficient for portions of CNTs (indicated by red dotted arrow), which is indicative of increase of strain shared on iCNTs (ε_i_). Other portions of CNTs only partially redshift and return to the free state quickly (indicated by blue dotted arrow). The evolution of ε_i_ with ε_f_ is shown in the contour plots, which depict the evolution of the parallel iCNTs within the (**C**) raw CNTF, (**D**) moderate DD-CNTF, (**E**) fully DD-CNTF, and (**G**) fully SD-CNTF, as well as the evolution of the perpendicular iCNTs within the (**F**) raw CNTF and (**H**) fully DD-CNTF. All the contour plots are normalized by the same scales and divided into zones (Roman numerals) based on the different behaviors of redshift. The white arrows are guides to the redshifts, with their corresponding load transfer ratio (ε_i_/ε_f_) being elucidated by the arrows’ slope. The maximum strains for iCNTs (ε_i_^*^) are also marked.

The ISSR spectrum is an amalgamation of every section on thousands of iCNTs within the laser spot, which can experience various strains. Along each CNT, the strain can be distributed unevenly. On the basis of the antenna effect of CNTs (*43*, *44*), we use ZZ/XX polarization configuration to detect the strain distribution of iCNTs parallel/perpendicular to the axis of CNTF (Fig. 7A). As shown in Fig. 7B, when CNTF strain (ε_f_) is gradually increased, sections in the spectrum start to redshift differently (red and blue dotted lines), from which the corresponding strain distribution among iCNTs (ε_i_) can thus be deduced (more details can be found in text S4). To highlight the evolution of ε_i_ with corresponding ε_f_, we accumulate these spectra of each CNTF into its contour plot (Fig. 7, C to H).

As the fiber strain increases to ε_f_ = 1.27% (zone i), the tubes parallel to the raw CNTF axis (ZZ configuration; Fig. 7C) exhibit a small broadening in the spectrum tail of low frequency (white solid arrow), while the middle and high-frequency sections change little (white dotted arrow). This implies that only a small proportion of CNTs share the strain on the fiber, while the others do not participate (*42*, *45*). When ε_f_ = 1.27%, the average ε_i_ can be deduced ~0.22% (calculation details can be found in text S4). We use load transfer ratio *LTR* ≡ ε_i_/ε_f_ as a figure of merit for load sharing. Thus, the average *LTR* (*$\$\bar{\mathrm{LTR}}\$$*) is only ~0.17, which indicates that the fiber strain ε_f_ is primarily a result of straightening of curled tubes, alignment of tubes toward the axis direction, or the relative slippage among tubes, rather than the strain increase on iCNTs. For larger strain, ε_f_ > 1.27%, the redshift reaches a plateau without any changes in the spectrum (zone ii). ε_i_ does not further increase and reaches a maximum, ε_i_^*^ ~ 1.65% (the corresponding LTR^*^ is ~0.49), indicating the occurrence of slippage between iCNTs. Approaching the failure point (ε_f_ ≥ 3.38%, zone iii), the broadening disappears (short white dotted arrows), which indicates that the remaining small portion of CNT bundles break and return to the initial state without any strain.

As a comparison, the fully DD-CNTF (Fig. 7E) shows a notable enhancement on the load sharing on the iCNTs. As load increases to ε_f_ = 0.97%, the entire peak of *G*′ mode redshifts without obvious broadening (white dotted arrow versus white solid arrow), which we referred to as zone i′. The corresponding $\$\bar{\mathrm{LTR}}\$$ increases to ~0.35 at ε_f_ = 0.97%. The redshift of the entire *G*′ mode indicates that a major portion of CNTs take the load with fiber. With greater strain, ε_f_ > 0.97%, there is no plateau of redshift in zone ii′. Instead, the spectrum splits into two groups: One group (indicated by the red arrow) continues to share the load as ε_f_ increases, while the other group (indicated by the blue arrow) gradually releases when strained, indicating the successive failure of thick bundles. For the former group, ε_f_ continues to increase and finally achieves ε_i_^*^ ~ 2.44% just before the rupture of CNTF, and the corresponding LTR^*^ can then be deduced as high as 0.89, indicating that a portion of CNTs synchronize with the fiber to take the load from the beginning until the fracture. The average *G*′ mode redshift rate per ε_f_ for this group is ~27.2 cm^−1^ %^−1^.We did not observe the zone iii strain releases to the initial state as occurred with the raw CNTF.

For the moderate DD-CNTF (η_CSA_ = 10.4% and η_*P*T_ = 0.5%; Fig. 7D), its behavior falls in between raw CNTF and fully DD-CNTF. The $\$\bar{\mathrm{LTR}}\$$ at the end of zone i is ~0.23. Like the fully DD-CNTF, zone ii′ appears with ε_f_ increases. ε_i_^*^ finally reaches ~2.07% just before the rupture of CNTF. The corresponding LTR^*^ is ~0.64. Without the Poisson tightening process, the fully SD-CNTF behaves similarly in zones i′ and ii′ (Fig. 7G), with $\$\bar{\mathrm{LTR}}\$$ ~0.29 at the end of zone i′, and LTR^*^ is ~0.83 when ε_i_^*^ ~ 2.09%. In zone iii, where ε_f_ approaches the rupture point, ε_i_^*^ cannot further increase, while the other portion of CNTs release to the initial state free of strain. In contrast, for the tubes perpendicular to the fiber axis (XX polarization), for both raw (Fig. 7F) and the fully DD-CNTFs (Fig. 7H), during the whole period, ε_i_ changes very little with the increase of *ε*_f_, indicating the redundancy of tubes perpendicular to the axis.

Collectively, the ISSR results for CNTFs with different enhancing processes enable insights into CNTFs and the double-drawing process, namely, (i) only a small portion of CNTs in the raw CNTF can take axial load; (ii) with only small strain on iCNTs, slippage will happen in raw CNTF; (iii) by drawing in CSA, a larger portion of CNTs can participate in load sharing upon initial loading; (iv) by drawing in CSA, higher strains on iCNTs are needed to lead to a slippage; and (v) Poisson tightening can further increase the strain capacity on iCNTs. The cumulative impact of these optimization from the double drawing elucidates the substantial increase in the breaking tenacity of the fully DD-CNTF.

## DISCUSSION

### Enhancing mechanism—the increase of effective bundles and the extension of CNT effective length in bundle

The improvement of properties after the saturation of alignment optimization, and the ever rising of $\$\bar{\mathrm{LTR}}\$$ and ε_i_^*^ after processing, indicates that additional factors must be included to develop a representative mechanism for CNTF loading. Misorientation of monomer units along the polymer chain has been recognized as the primary factor that leads to the reduction of stiffness of many synthetic fibers (*46*). The tensile Young’s modulus of fibers is commonly estimated by the appropriate average of the moduli of all monomers along the axis (*24*). However, the assumption implies that all monomers evenly participate within a fiber, which is not suitable for a fibrillar assembly like CNTF. The movement and deformation of iCNTs within CNTFs are not all affine. Instead, under load, a tensioning line frequently appears from the disordered network in raw CNTFs, which indicates the stress concentration, also the only portion of CNTs bearing the load (detailed analysis can be found in text S5).

**Fig. 8.**
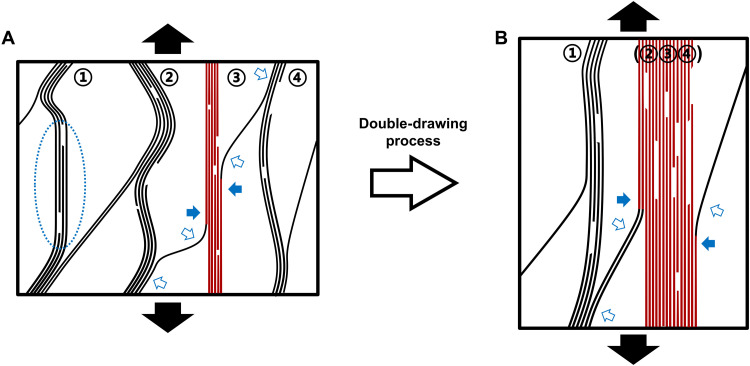
The two-dimensional schematic graph of the optimization on bundles being effective and the effective length of tubes within a bundle after the double-drawing process. (**A**) In the disordered CNT network, tubes (lines) aggregate as bundles (marked as ① to ④). Within the schematic cell under vertical stretching, only the shortest bundle connecting the vertical surfaces takes load, i.e., being effective (painted in red). Tubes outside the loaded bundle remain idle despite portions being oriented parallel to the load (circled with blue dotted circle). For tubes that link to multiple bundles, only the length section of tubes attached to an effective bundle take load (solid blue arrows), while the external length section remains idle (hollow blue arrows). (**B**) With the double-drawing process, the crumpled iCNTs are straightened as the shortest pathway and more tubes are compressed into a large bundle (②③④), extending the effective length section.

As shown within a simplified CNTF cell (Fig. 8A), if the load is exerted on the vertical surfaces of the cell (along axis), there is no medium between CNTs to transmit the load; thus, only the shortest CNT bundle is loaded (bundle ③, the red lines). While alignment analysis by WAXD shows a substantial alignment, the portion of idle CNTs cannot be determined. Therefore, for the fibrillar structures, the straightening of bundles is a more indicative metric for fiber strength than orientation factor. After the drawing in CSA, more crumpled tubes and bundles straighten [②③④] and become effective to link the “shortest” distance (the red lines in Fig. 8B). They jointly participate in sharing the load after the initial stretching. More correlations between this factor to the optimization of ISSR results and mechanical properties can be found in text S5.

Nevertheless, only accounting for the increased fraction of load-bearing bundles in network does not fully explain the much higher ε_i_^*^ for the DD-CNTF. As the ε_i_^*^ appears immediately before failure, the much-improved tenacity for the fully DD-CNTF must therefore be considered. For the elastic interface (static friction), the stress exerted on iCNTs *σ*_i_ = *Y* ε_i_ is balanced by the friction from the surrounding tubes, where *Y* and *ε*_i_ are the tube’s Young’s modulus and strain, respectively (*47*). When σ_i_ increases to the critical value σ_i_^*^, slippage will occur and the elastic interface will begin to deform plastically. Immediately before slippage, σ_i_^*^ *A*_t_ *= f*_s_ *L*_eff_, where *A*_t_ is the cross-sectional area of the tube, *f*_s_ is tube’s maximum static friction coefficient per unit length, and *L*_eff_ is the effective length of tube that shares the load (friction). Thus, σ_i_^*^ is the maximum value for *σ*_i_ and the corresponding *ε*_i_^*^ can be deduced by

$$\varepsilon_{i}^{*}=\frac{\sigma_{i}^{*}}{Y}=\frac{\;f_{s}L_{\mathrm{eff}}}{YA_{t}}$$ (1)

In a fibrillar structure, particularly raw CNTF [entire length of iCNTs *L* ~ 100 μm (*1*)], tubes within the hierarchical network only partly align with any specific bundle and may be incorporated into many bundles. Because the load can only be transmitted through the coupling between adjacent tubes, only the tube section attached to an effective bundle can participate in sharing the load (the red lines as indicated by solid blue arrows in Fig. 8), i.e., *L*_eff_ << *L* (Fig. 1, B and C). With the double-drawing process, a longer length of tubes aggregates into the effective bundles ([②③④] in Fig. 8B), with *L*_eff_ approaching *L*. Consequently, ε_i_^*^ needed to activate the slippage also increases, which delays the failure of DD-CNTF and improves the tenacity (discussed further in text S5).

Although the conduction pathway for heat is not essential to be shortest, the thermal conduction benefits when more bundles and larger fraction of tubes become effective. The junction boundaries between bundles are likely the first-order contribution to thermal resistance (*48*), which become sparser along the fiber axis after the double drawing. With the increase of *L*_eff_ and alignment of adjoining bundles, the joint length and interfacial area between tubes are extended, improving the thermal conductance through the corresponding junctions. In addition, we attribute the increase on the work of rupture after Poisson tightening to the extension of *L*_eff_ and the enhanced interaction between adjacent tubes after the removal of CSA, both of which require more energy to slide tubes during stretching.

In summary, we have developed a technique to enhance CNTFs with crystalline defects (*I*_G_:*I*_D_ ~ 5) and greater *LD* (0.42 tex) by successively drawing the raw direct-spun CNTFs within CSA and chloroform at ambient temperature and pressure, which rearranges the CNT bundles, densifies the fibers, and removes the residue CSA via Poisson tightening and rinsing. With full double drawing, CNTFs reach a tenacity of 3.30 N tex^−1^ (4.60 GPa), Young’s modulus of 130 N tex^−1^, rupture work of 70 J g^−1^, electrical conductivity of 10700 S cm^−1^, and thermal conductivity of 354 W m^−1^ K^−1^. The high performance with low-crystallinity CNTs demonstrates that the limiting feature of CNTFs is their mesoscale arrangement rather than atomic-scale vacancies. The resulting simultaneous optimization properties result in an attractive overall performance, continuing the impressive line of improvement seen within CNTFs worldwide in recent years. We anticipate further advancement in material properties using the double-drawing technique, which is applicable to other CNT ensemble, including forests, films, fibers, and aerogels.

In addition to the known dependence of properties on CNT alignment and stacking, new evidence of the load transfer coefficient on iCNTs highlights the importance of (i) straightening of CNT bundles, which increases the proportion of effective bundles jointly sharing the load, and (ii) the higher barrier of slippage activation within bundles, which originates from the effective tube length increase within effective bundles. The comprehensive improvement of CNTF properties enables long-sought applications of advanced load-bearing fibers that can simultaneously dissipate heat, shield impacts, or dissipate electrical charge. Comprehensive understanding, advancement of these multitude of mechanisms, and resulting properties are necessary to manifest bulk CNTF properties that approach the horizon of properties offered by iCNTs.

## MATERIALS AND METHODS

### CNTF preparation

Continuous CNTFs were fabricated using the floating catalyst method (*49*, *50*) and supplied by Tortech Nanofibers Ltd. The produced CNT aerogels from a tube furnace were mechanically pulled out, densified by acetone, and spun continuously winded. Although a small tension force is applied during the spinning process to obtain a preferential alignment along the fiber axis, the anisotropic ratio is always within 0.85 (*51*).

### Enhancing CNTF with the double-drawing process

#### Immersing and first drawing

The raw CNTF is fixed at its lower end inside a dropping funnel, and its upper end is fixed on a spin rotor. CNTF is straightened out but without a pretension. After being immersed in CSA, CNTF is first drawn to a specific ratio (η_CSA_).

#### Poisson tightening

After the immersing solvent is changed into chloroform, CNTF is immediately further drawn by η_PT_.

#### Rinsing

After the double-drawing processes, CNTF is successively rinsed in water and acetone and finally vacuum-dried.

### *LD* measurement

The *LD* is measured on the basis of direct single-fiber weight determination following ASTM D1577-07(2018) OPTION B. The weight of a CNTF with length ~100 mm is measured with a Sartorius SE2 Ultra-micro balance. We find it worthy to notice the importance of accurate measurement of *LD* because of the susceptibility of fiber’s tenacity to *LD*. The frequently used vibroscopic method in reports is abandoned here because of the potential serious underestimation of *LD* if the stiffness correction was overlooked [ASTM D1577-07(2018) OPTION C—the standard for the vibroscopic method]. More discussion can be found in text S2.

### Tensile test

CNTFs are tested using the single-fiber testers [Textechno FAVIMAT with load cell of 210 cN and delicately aligned clamps (4-mm hard rubber), force resolution of ~0.0001 cN, and displacement resolution of 0.1 μm]. CNTFs are tested using a gauge length of 10 mm, a stretching speed of 1 mm min^−1^, and a pretension of 0.1 cN tex^−1^. Every sample is tested for three specimens to guarantee the repeatability of results. Stretching speeds of 0.2, 2, and 5 mm min^−1^ and gauge length of 20 mm have been tried to generate similar results.

### Thermal and electrical conductivity measurement

The thermal conductivity of CNTF along the fiber axis is performed using a homemade measuring apparatus based on a self-heating method (*52*). The electrical conductivity of CNTFs along the fiber axis is measured in air at room temperature (1 atm; 25° to 27°C; relative humidity, 40 ± 3% RH) by a homemade testing stage using the four-electrode method and steady-state method (*53*).

### In situ stretching Raman

The suspended CNTFs are end-fixed onto a manual stretching stage to detect the Raman signal with HORIBA HR800 micro-Raman spectroscopy. We excite the Raman *G*′ mode with linearly polarized laser and only collect the scattered radiation in the parallel polarization with a Glan polarizer so that only iCNTs with their axes close to parallel with the laser polarization can be detected. For the ZZ/XX configuration (*54*), the polarizations of both incident and scattered photons are parallel/perpendicular to the axis of CNTFs, offering the strain distribution of CNTs along/normal to the fiber axis.

### Wide-angle x-ray diffraction/small-angle x-ray scattering

CNTFs with different processing are studied using a small and wide-angle diffractometer (Molecular Metrology SAXS system) equipped with a sealed microfocus tube (MicroMax−002+S) emitting Cu Kα radiation (wavelength of 0.1542 nm), two Göbel mirrors, and three pinhole slits. CNTFs with a diameter of 18 to 50 μm were suspended onto a holder perpendicular to the beam and measured at ambient temperature. All the raw data are analyzed by SAXSGUI. For data analysis of WAXD, the sharp equatorial reflections at *q* ~ 1.8 Å^−1^ correspond to the scattering from (002) reflection of the interlayer spacing of a few walled CNT and to a higher-order reflection of the hexagonal packing of parallel CNTs. Both possibilities are due to the planes perpendicular to the CNT axis. To obtain the azimuthal profile of (002) scattering, the intensity is integrated in the range of 1.6 to 1.9 Å^−1^. With the increase of the alignment, two peaks emerge in the azimuthal profile around the preferred alignment, from which FWHM is obtained. For data analysis of SAXS, the integrating range is 0.04 to 0.1 Å^−1^ to obtain the azimuthal profile of scattering.

### Other characterizations

The cross sections of CNTFs are fabricated using FIB (FEI Helios 600i). The cross section is first cut using Gallium ions with current of 9 nA (30 kV) and then finely polished under current of 0.79 nA, after which the SEM of cross sections is conducted by electron beam of FIB. The SEM for other CNTFs is conducted on TESCAN MIRA3. High-resolution transmission electron microscopy (HRTEM) is conducted on an FEI Talos F200X TEM working under 80 kV to reduce the damage to CNTs.

## Supplementary Materials

### This PDF file includes:

Texts S1 to S5 Figs. S1 to S12 Tables S1 and S2 References

## Notes

View/request a protocol for this paper from *Bio-protocol*.
