# Supplementary material for: Simultaneously enhanced tenacity, rupture work, and thermal conductivity of carbon nanotube fibers by raising effective tube portion
Source: Sci Adv. 2022 Dec 14;8(50):eabq3515. doi: 10.1126/sciadv.abq3515 (PMC9750159; doi:10.1126/sciadv.abq3515)
Supplement: Supplementary file 1 — Texts S1 to S5 Figs. S1 to S12 Tables S1 and S2 References [file sciadv.abq3515_sm.pdf]

Science Advances
AAAS

Supplementary Materials for

Simultaneously enhanced tenacity, rupture work, and thermal conductivity of carbon nanotube fibers by raising effective tube portion

Xiao Zhang *et al.*

Corresponding author: Adam Boies, amb233@cam.ac.uk; Sishen Xie, ssxie@iphy.ac.cn; Michael De Volder, mfld2@cam.ac.uk; James Elliott, jae1001@cam.ac.uk

Sci. Adv. **8**, eabq3515 (2022)
DOI: 10.1126/sciadv.abq3515

This PDF file includes:

Texts S1 to S5
Figs. S1 to S12
Tables S1 and S2
References

Supplementary Text

S1. Experiment details on the Double-Drawing process

The raw CNTFs are continuously fabricated with a CVD reactor at 1100−1200 °C using the floating catalyst method, and supplied by Tortech Nanofibers Ltd. In the fabrication process, a preheated feedstock consisting of toluene (carbon source), ferrocene (catalyst precursor), thiophene (promoter), and hydrogen (carrier gas) with flow rates of 1−2 g h^{-1}, 100−400 mL min^{-1}, 10−20 mL min^{-1}, and 1000−2000 mL min^{-1}, respectively, was injected into a reactor to form CNT aerogels continuously. The CNT aerogels were mechanically pulled out, densified by acetone, and spun continuously with a motorized winding system. Based on the above methods, the CNTFs can be produced at a rate of 20–30 m min^{-1} (i.e., 1.2-1.8 km h^{-1}) (*8*), which had been further increased to >50 m min^{-1} (i.e., >3.0 km h^{-1}) by Vilatela et at. (*9*). A similar direct spinning CNTFs product, naming Miralon®, is reported to be produced at a rate of 5 kg h^{-1} (~500 km h^{-1}) with a cost of ~$100 kg^{-1} (*10*).

Although a small tension force is applied during our spinning process to obtain a preferential alignment along fiber axis, the anisotropic ratio is always within 0.85. The raw CNTFs, normally with linear density ~0.5 tex and I_G:I_D ~5.3 are used for the subsequent processing.

To enhance the raw CNTFs, the fiber is firstly fixed at its lower end inside a dropping funnel, and its upper end fixed on a spin rotor (**Figure S9**a). After being immersed in chlorosulfonic acid (CSA, Sigma-Aldrich®, 571024-100G) for 30 s, CNTF is drawn to a specific ratio (η_{CSA}) at a rate of 1 mm s^{-1}. To fully draw the CNTF, the maximum ratio η_{max} is used, which is slightly less than the failure ratio η^* in CSA. The drawing rates from 0.1 mm s^{-1} to 10 mm s^{-1} have been tried without generating obvious difference.

After the immersing solvent is exchanged for chloroform, the drawn CNTF is immediately further drawn by η_{PT} = 0.5%. When the CNTF is only immersed in chloroform (Sigma-Aldrich®, C2432-1L), pimples of CSA always appear on the surface of drawn CNTF (**Figure S9**b-c); this is because the closely compacted tubes on the outer fiber layer hinders the diffusion of the remaining CSA from the inner layers. The remaining CSA keeps screen the vdW forces through which the load transfer. Therefore, the second drawing process in chloroform is necessary. The pimples disappear during the second drawing process (**Figure S9**d).

After the Double-Drawing processes, the CNTF is successively rinsed in water and acetone, and finally vacuum dried at 200 °C for 2 hours.

We find the strong dependence of η^* on linear density (*LD*) of CNTF. For example, for CNTFs with *LD* ~0.47 tex, η^* ~28%; and for CNTFs with *LD* ~5.38 tex, η^* ~125%. With the thickening of the CNTF, larger η^* is needed to straighten all the tubes across the thickness. This conclusion is also applicable for other direct spun CNT assemblies, like mat and film.

In our experiment, all the raw CNTFs are used directly without any purification. Although by heating and acid treatment, impurities like catalysts and amorphous carbon can be removed, we find that the inevitable defects generated during the purifying process result in the excessive drawing at variable points and the degradation of the draw-ratio on other part.

S2. Precise Determination of Linear Density for CNTFs

The accuracy of tenacity (specific strength) value for micro-fibers heavily relies on the accurate measurements of linear density (*LD*).

Researchers usually used the Vibroscopic methods to deduce *LD* of CNTFs. By introduce transverse vibration with oscillatory force, *LD* can be deduced by finding the fundamental resonance frequency (f_0) of the fiber under known conditions of gauge length (l) and pre-tension tension (F_{PT}) with following equation (*55*):

$$LD = \frac{F_{PT}}{4{f_0}^2 l^2} \tag{S1}$$

However, Equation (S1) only works well under the assumption that tested fibers are perfectly flexible. However, the assumption can cause problems for CNTFs. As mentioned in ASTM D1577−07(2018) OPTION C, correction must be made on f_0 to account for the "stiffness effect" (*24*, *55*, *56*), following the equation below:

$$LD_c = \frac{F_{PT}}{4f_{oc}^2 l^2}(1 + 2\alpha + 5.47\alpha^2) = \frac{F_{PT}}{4f_{oc}^2 l^2}(1 + \delta) \tag{S2}$$

Here, f_{oc} is the measured apparent fundamental resonant frequency, stiffness factor $\alpha \equiv (4EI/l^2 F_{PT})^{1/2}$, and E is the Young's modulus of the fiber, I is the inertia moment of the fiber about the neutral axis. For a circular cross-section fiber with diameter, $I=\pi d^4/64$. And $\delta \equiv 2\alpha + 5.47\alpha^2$ is the increase factor for *LD* because of the "stiffness effect". Thus, the real tenacity consequently should be $1/(1+\delta)$ lower compared to that without considering "stiffness effect".

Therefore, δ becomes non-negligible if fibers possess (a) high Young's modulus, or (b) relative short gauge length, or (c) being tested with low pre-tension. As to the post-processed CNTFs with high modulus and commonly used testing parameters, the measured *LD* always deviates away from that based on perfectly flexible string model. Based on some typical reported data, δ deduced can be as high as 33%, which is unreasonable to be ignore. On the other hand, if tested with high pre-tension, the CNTFs may undergo plastic deformation.

Considering the above factors, we choose the Direct Single-fiber Weighing method as the reliable measurement of *LD* following ASTM D1577−07(2018) OPTION B, because the accuracy is only dependent on the accuracy with which the fibers can be length measured and weighed.

S3. Surface morphology and tenacity of CNTFs only immersed in CSA

As shown in Fig. 4c, by only immersing in CSA, the CNTF gains ~40% increase in tenacity. Moreover, after CSA processing, CNTFs always contain short and straight CNT bundles on the fiber surface (Fig. 4d). We believe these straight bundles originate from the high stiffness and persistence length of iCNTs and their bundles which behave similarly to semiflexible filaments, with their persistence length much larger than the pristine curved diameter formed in raw fiber (*29*, *33*). When the athermal structure of CNTF "melts" upon immersion in CSA, the curved iCNTs spontaneously rearrange towards an equilibrium organized structure with improved properties, while dangling iCNTs on the surface rearrange into a rod-like shape as observed.

S4. Experiment details on the In-Situ Stretching Raman

Monitoring the redshift of Raman mode is often the clearest and simplest characterization to detect strain on iCNTs.

During the In-Situ Stretching Raman (ISSR) characterization, the suspended CNTFs are ends fixed onto a manual stretching stage to detect the Raman signal with HORIBA HR800 micro-Raman spectroscopy. We excite the Raman G' mode with linearly polarized laser with power on sample ~4.9 mW. Because the redshift level is proportional to the strain. Among the Raman modes, the two phonon processes, such as the G' mode, possess a higher redshift rate (~70 cm^{-1} $\%^{-1}$), which increases the accuracy of the strain distribution measured. Moreover, the insensitivity of G' mode on the types of CNTs also facilities the deducing of strain.

Within CNTFs, the diameter of CNTs is ~10 nm. The laser spot size (~2 um) covers thousands of tubes and length in micron scale in each tube. Therefore, the Raman scattering signal gives the strain distribution among these thousands of tubes and along each iCNTs. Because strain, which is proportion to the content of redshift, is unevenly distributed inter and intra tubes, the redshift signal detected gives the strain distribution normalized by volume. Because the fiber is isotropic across the depth (as verified by cross-section cut by FIB), the results of ISSR offers the overall description of the fiber. Moreover, for iCNTs, the linewidth of Raman mode will not increase even with a strain at least 4% (*41*), thus, the broadening of Raman peak with stretching can be converted to the broadening of strain distribution instead of the linewidth broadening of Raman mode itself. Therefore, we can use the collective signal to analyze the distribution of strain among CNTs.

Here, with a Glan Polarizer, we only collect the scattered radiation in the parallel polarization with the laser, so that only iCNTs with their axis nearly along the laser polarization can be detected. For the ZZ/XX configuration (*54*), both incident and scattered photons detected parallel/perpendicular to the axis of CNTFs, offers strain distribution of CNTs along/normal to the fiber axis. Additionally, we have not noticed the relaxation of strain during the ISSR experiment. At least 95% of force retention within 10 mins is verified by the strain relaxation experiment (Fig. S8). The force retention is sufficient for us to complete the ISSR characterization.

S5. Details on the enhancing mechanism

- **Why the raw CNTFs are weak on mechanical properties:**

When a raw CNTF is under load, tensioning lines frequently appear from the disordered iCNTs network in raw CNTFs, which indicates the stress concentration. The tensioning lines are also the small load-bearing portion of CNTs as indicated by ISSR (Fig. 7c). Around the tensioning line, the slippage and failure happen first. This is because the tensioning line, as the shortest pathway, is the first region of the material to take the load and alone.

In the fibrillar structure of CNTFs, the rupture procedure under increasing load proceeds with the shortest bundle breaking first, then the second-rated shortest bundle takes the increased load, and so forth. Eventually, as the major distribution of bundle lengths is approached and broken, there will be insufficient bundle left to support the load, and catastrophic failure will occur. Because in raw CNTFs, at any time of stretching, only a small portion of bundles are shortest within the disordered network. During the whole stretching process, the bundles just successively break with a unit of small portion of them. Therefore, the tangent modulus at any time of stretching is low.

- **1st Enhancing mechanism: the increased proportion of load-bearing bundles.**

As shown within a simplified CNTF cell (Fig. 8a), if the load is exerted on the vertical surfaces of the cell (along axis), because there is no medium between CNTs to transmit the load, only the shortest CNT bundle is loaded (bundle ③, the red lines). The curved bundles (bundle ①②④) do not substantially contribute to bearing the load, despite portions of them being oriented in the vertical direction (circled by blue dots line). As observed in the ISSR, a substantial proportion of CNTs do not share the load, particularly for the raw CNTF with disordered microstructure.

After the drawing in CSA, more crumpled tubes and bundles are straightened and become effective to link the "shortest" distance (the red lines in Fig. 8b). They jointly participate in sharing the load after the initial stretching, indicated by the soaring of initial modulus (Fig. 4b, d). This optimization is also verified by the entire redshift of G' mode in Zone i' (Fig. 7e and g) and the larger portion of tubes further redshift in Zone ii'. As the load continues to increase, effective bundles fail successively, decreasing the modulus monotonically (Fig. 4b). The failed bundles return to the initial state free of strain, as observed in the redshift release in Zone ii' (Fig. 7b and e blue dotted arrow).

- **2nd Enhancing mechanism: the extension of effective length of tubes in load-bearing bundles.**

Only accounting for the increased fraction of load-bearing bundles in network does not fully explain the much higher final/maximum strain on iCNTs, i.e., ε_i^* for the DD-CNTF. Another factor must be considered for the mechanism, particularly considering the maximum strain turns up just before the failure. The much-improved tenacity value for the fully DD-CNTF must link to this factor.

We attribute the optimization of ε_i^* to the effective CNTs length increase within load-bearing bundles, which results in a higher activation barrier for slippage. As found recently by Yakobson et al, the product of the tube length and the friction coefficient between tubes is positively correlated with the tensile properties of a bundle (*47*). For the elastic interface (static friction), the stress exerted on iCNTs $\sigma_i = Y\varepsilon_i$, is balanced by the friction from the surrounding tubes, where Y and ε_i is the tube's Young's modulus and strain, respectively. When σ_i increases to the critical value σ_i^*, slippage will occur and the elastic interface will begin to deform plasticly. Immediately prior to slippage, $\sigma_i^* A_t = f_s L_{eff}$, where A_t is the cross-sectional area of the tube, f_s is tube's maximum static

friction coefficient per unit length, and L_{eff} is the effective length of tube that sharing the load (friction). It is easy to find that σ_i^* is the maximum value for σ_i, because L_{eff} will shrink as the tube-tube contact area drops during relative slipping. Therefore, the corresponding ε_i^* can be deduced by:

$$\varepsilon_i^* = \frac{\sigma_i^*}{Y} = \frac{f_s L_{eff}}{Y A_t} \tag{S3}$$

Previous models often contain an implied assumption that the entire lengths of tubes (*L*) are attached to the bundle, i.e., $L_{eff} = L$. However, in a fibrillar structure, particularly raw CNTF, tubes within the hierarchical network only partly align with any specific bundle, and may be incorporated into many bundles. Particularly, for the CNTF used in this work, tubes produced by FCCVD have long lengths, ~100 µm.(*1*) Because the load can only be transmitted through the coupling between adjacent tubes, only the tube section attached to an effective bundle can participate in sharing the load (the red lines as indicated by solid blue arrows in Fig. 8), i.e., L_{eff} is always smaller than *L*. In contrast, the dangling CNTs or fraction of CNTs attached to an ineffective bundle (hollow blue arrow) do not transmit load, even though these portions may belong to the same tube. As observed in Fig. 1b–c, in most cases for raw CNTF, $L_{eff} << L$.

With the Double-Drawing process, the crumpled tubes are extended by drawing and squeezed together by Poisson Tightening, forming bundles with large diameter (red aggregated bundle [②③④] in Fig. 8b, Fig. 1d–e and Fig. 2a). Thus, longer length of tubes aggregates into the effective bundles, with L_{eff} approaching *L*. As per Equation (1), an increase in L_{eff} proportionally increases ε_i^* needed to activate the slippage, which delays the failure of DD-CNTF and improves the tenacity.

- **Why the tenacity can still show an upward trend of tenacity even after the saturation of alignment:**

For a fibrillar assembly, like CNTFs or cotton yarns, only the shortest constituents within a cell unit can participate in sharing the load. During the process of the curled tubes being drawn to the shortest constituents, the alignment may be synergistically improved but not have to be necessary. For example, for the moderate Double-Drawn CNTFs (with draw-ratio ~ 14%), the alignment is nearly saturated as characterized by WAXD and SAXS. However, still only small fraction of bundles is taking the load, because the others are not the shortest.

With further drawing in CSA, the load-bearing tubes will slide relative to other tubes because of the low vdW forces constraint. Increased bundles will sooner or later become the shortest bundles within a cell unit. When approaching η^*, CNTFs' failure draw-ratio in CSA, most of the bundles become the shortest ones among unit cells. After the Poisson Tightening and washing, these bundles can bear the load jointly at the initial state. During this further drawing step, the alignment optimization is not prominent.

It also worth noting that alignment saturation does not mean perfectly aligned. Because of mutual constraint from adjacent tubes, many bundles cannot be perfectly aligned even till the end, but they can still share the load. The alignment instead cannot guarantee tubes to share the load. Only the case of 100% tubes being perfectly parallel with the fiber axis, the alignment and being shortest are equivalent. However, we need to supplement that after the constituents become the shortest, the orientation of them determines their contribution to the load.

The factor of higher barrier of slippage activation within bundles also contributes. As mentioned in the main text, only the section of tubes attached on these load-bearing bundles

can contribute. With further drawing and the necessary Poisson Tightening, more tubes slide along the load-bearing bundles, longer of them finally attach onto these load-bearing bundles and become effective. Because the barrier of slippage activation is proportion to the effective section length of tubes, as mentioned in Eq. 1, the tenacity becomes higher. During this process, the alignment optimization is not prominent.

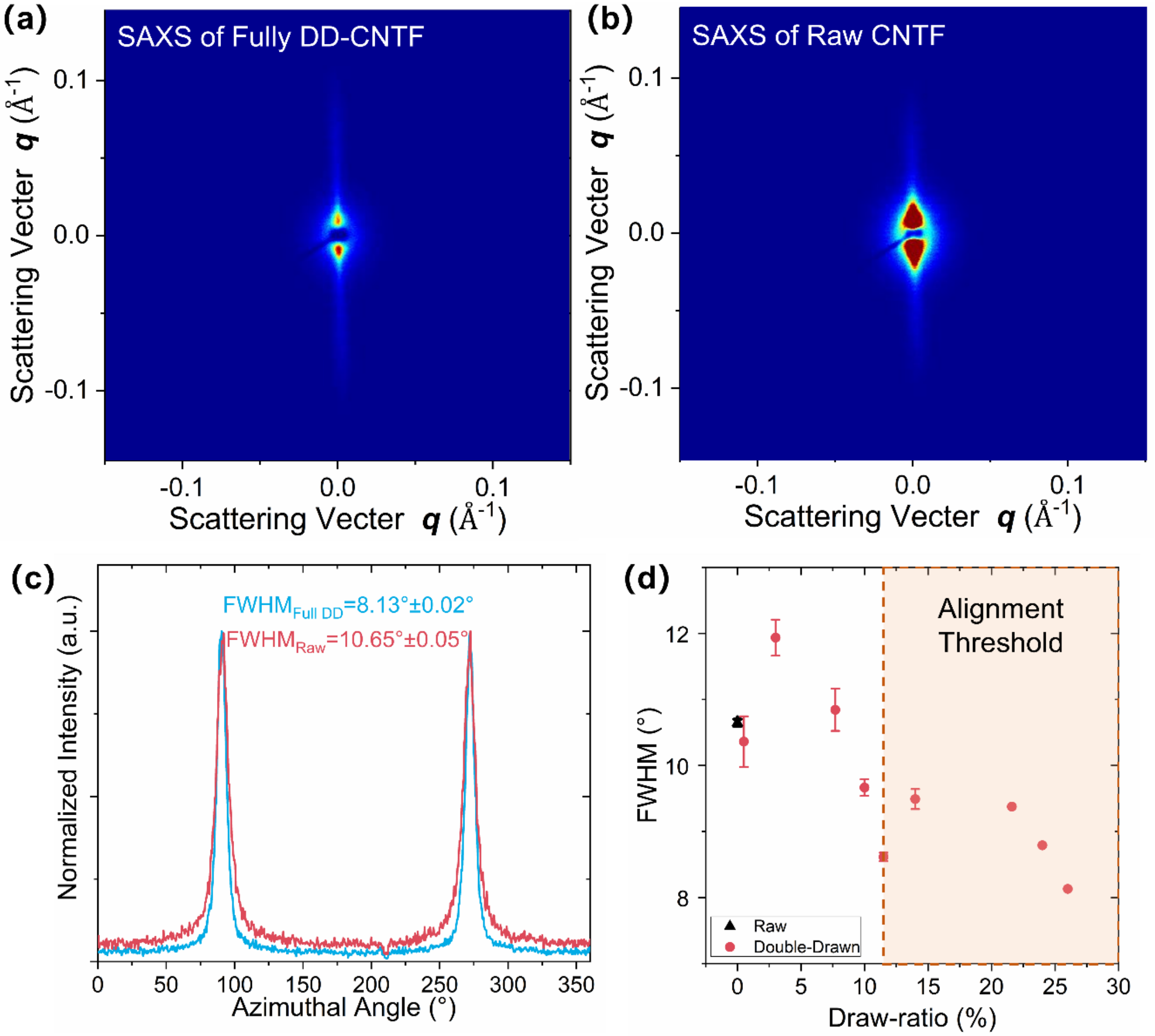

Fig. S1. SAXS pattern evolution of CNTFs with the increase of draw-ratio. (a–b) the SAXS patterns from the suspended fully DD-CNTF and raw CNTF. (c) The azimuthal profiles of the SAXS intensity illustrate peaks from the preferred alignment in CNTF. (d) The evolution of alignment peak in SAXS azimuthal results of suspended CNTFs. Like the (002) peak of WAXD azimuthal profile, the alignment of iCNTs within CNTF indicated by the FWHM of peaks in SAXS azimuthal profile decreases with the gradually increase of draw-ratio but seems to saturate after threshold level of drawing (12%).

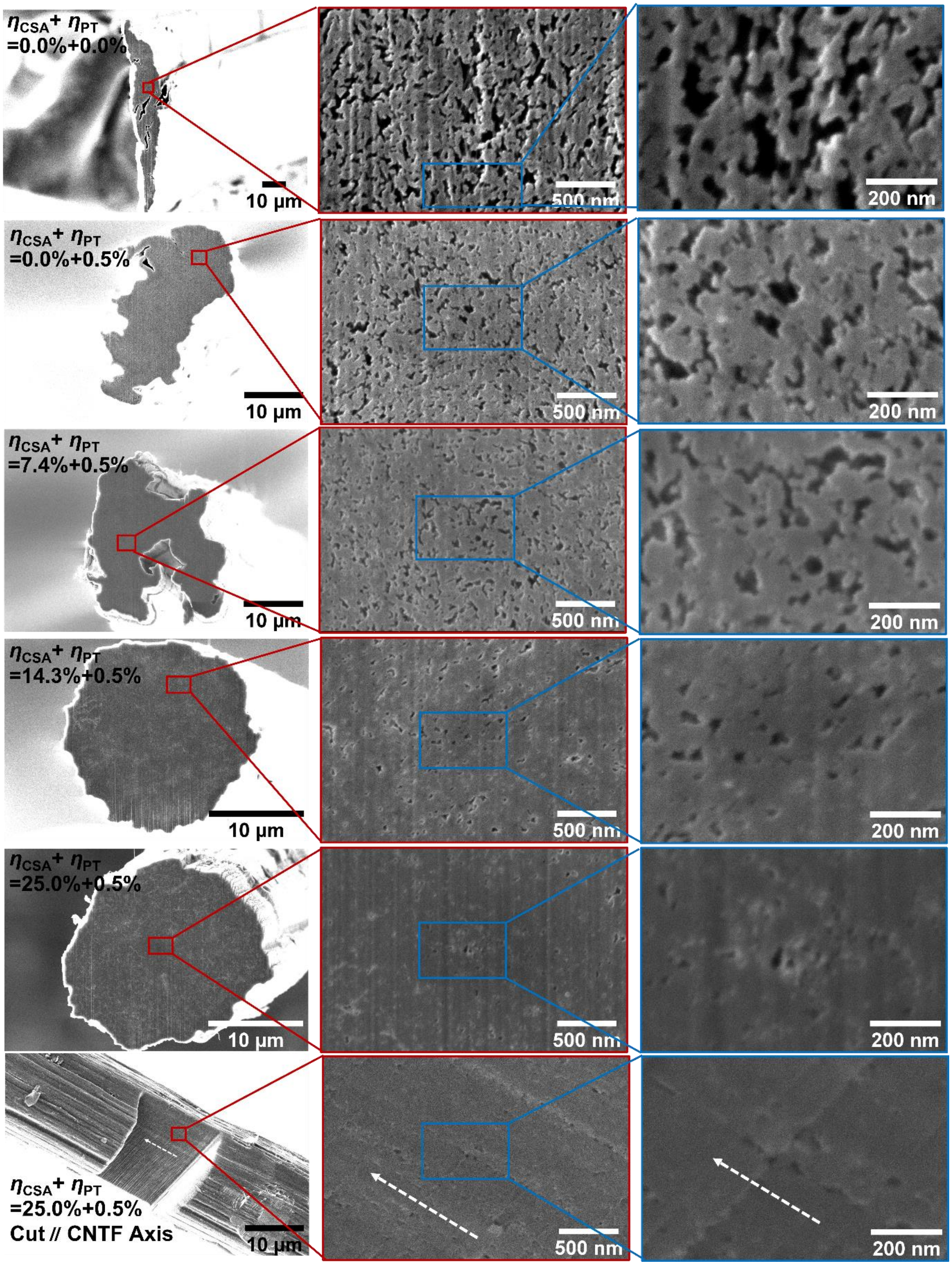

Fig. S2. The evolution of the porosity within the CNTFs after the increase of drawing. On the cross-section cut with FIB, the trend is obvious that the porosity monotonically decreases with the increase of draw-ratio. We further checked the cross-section parallelly cut along the fiber axis (last row), the stubborn voids are all in a configuration of beads-chain along fiber axis (white arrow). Because the area surrounded by the voids is an indicator of the cross-section of a bundle, the ever

increasing of solid area also illustrates the thickening of bundles, as mentioned in the Microstructure section of main text. The bright lines in images come from the Curtain Effect originated during the FIB cutting. We remove the bright lines in the last row by image processing method (*57*) to highlight the voids configuration.

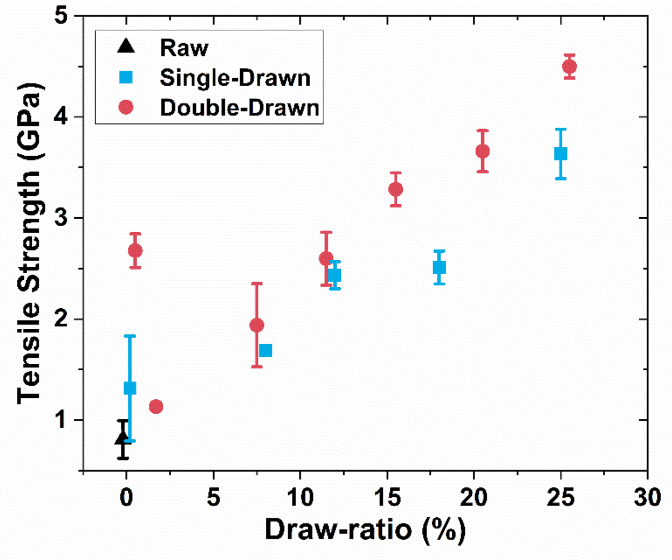

Fig. S3. The evolution of the CNTFs' ultimate tensile stress (strength) with the rising of draw-ratio. All the strength values are calculated by dividing the breaking force with the apparent cross-sectional area measured with SEM. For the fully DD-CNTF, the strength is improved to 4.50±0.11 GPa, compared with 0.81±0.19 GPa for the raw CNTF.

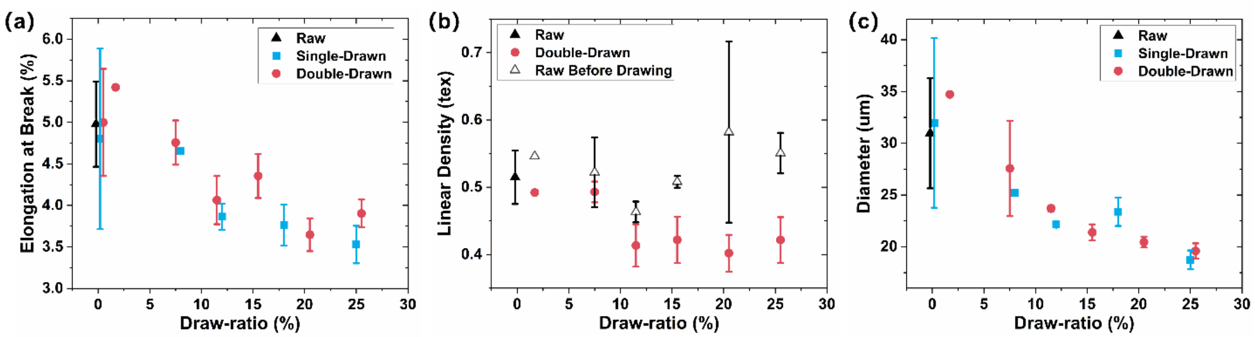

Fig. S4. The evolution of the CNTFs' elongation at break (ductility), linear density and diameter with the increase of draw-ratio. For both Double-Drawn (DD) and Single-Drawn (SD) CNTFs, the ductility keeps decreasing with the draw-ratio. Although exerted extra drawing (Poisson Tightening), DD-CNTFs are not sacrificed on ductility when compared with SD counterparts. The large error bars for the raw and slightly drawn CNTFs come from the difference between fiber samples. The error bars are reduced with drawing. (b) Changes on linear density from raw CNTFs to the corresponding DD-CNTFs. The linear density of the raw CNTF fluctuate along the axis on metre scale because of the fluctuation of CNTFs production facility. (b) The evolution of the CNTFs' apparent diameter (measured by SEM) with the increase of draw-ratio. For both DD and SD-CNTFs, the diameter keeps decreasing with the draw-ratio. The larger error bars for raw CNTFs originate from the more irregular and porous cross-section.

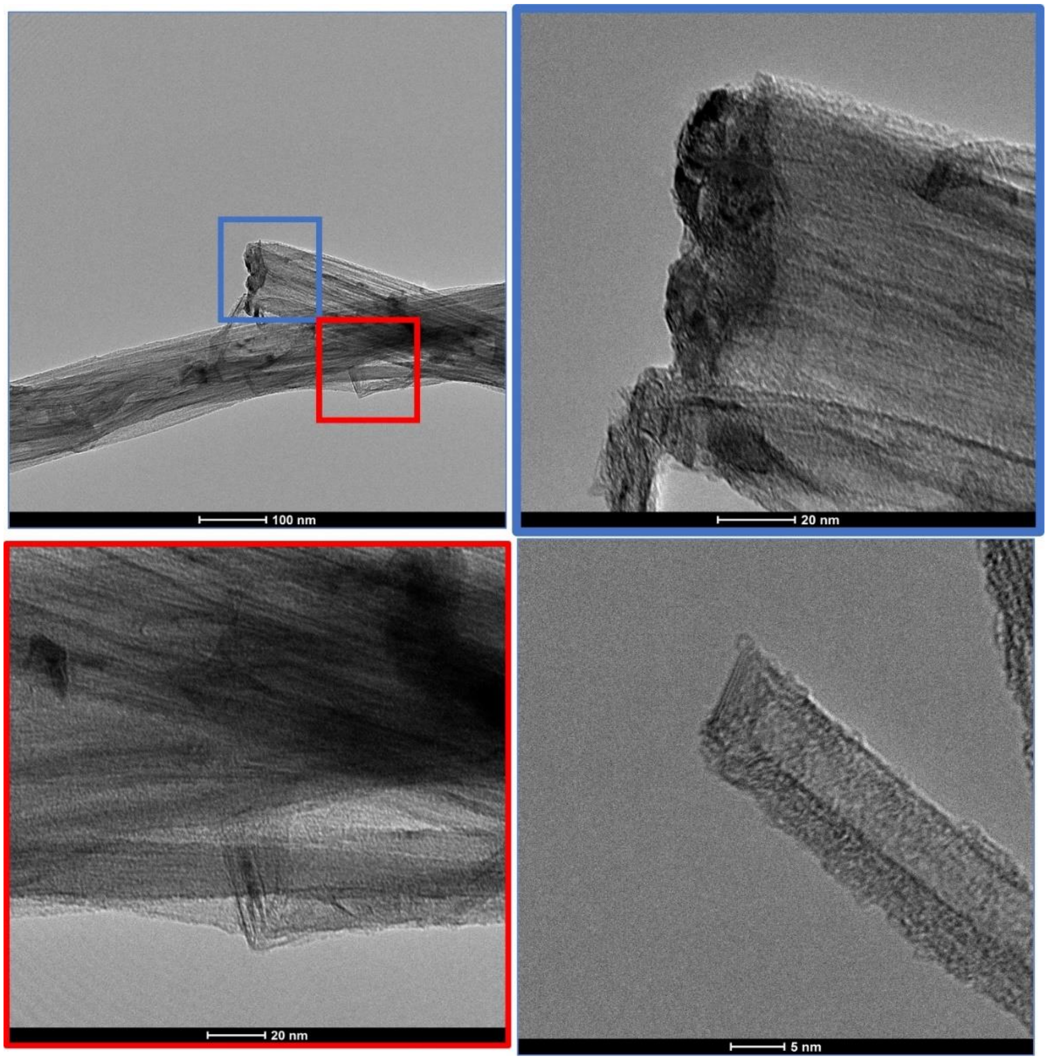

Fig. S5. With HRTEM characterization, all the cross-sections of bundles we found are collapsed ones instead of the deformed irregular ones. We think it may come from the radial compressing during the Double-Drawing process. The collapsed cross-section also has a positive impact on f_s by maximizing the contact area between tubes, which can further enhance the bundle (*50*).

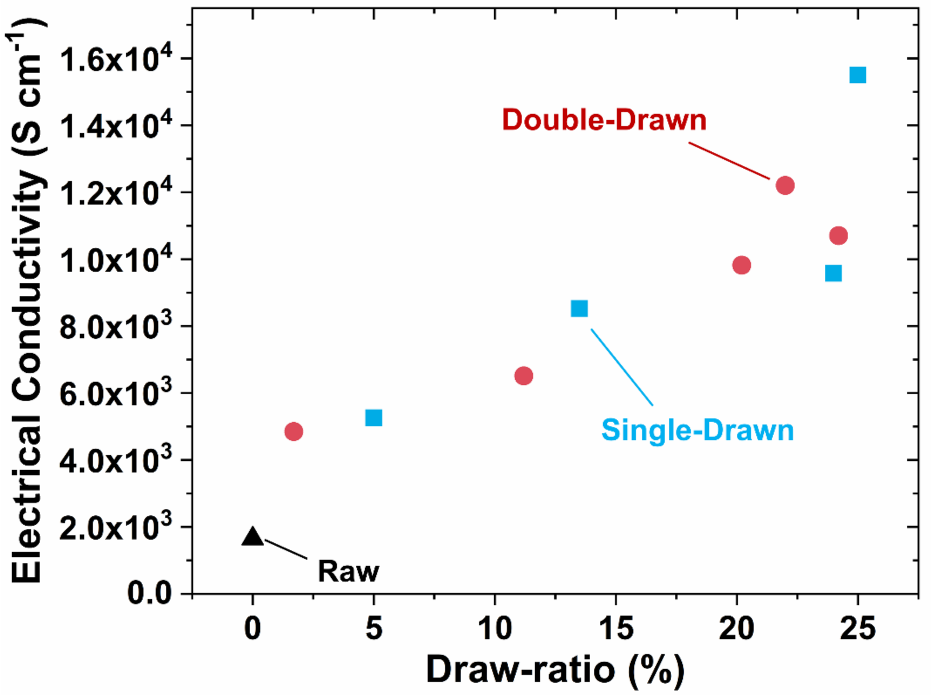

Fig. S6. Evolution of electrical conductivity with the increase of draw-ratio. The electrical conductivity substantially increases from 1650 S cm^{-1} of the raw CNTF to 10,700 S cm^{-1} of the fully DD-CNTF, and 15,500 S cm^{-1} of the fully SD-CNTF. However, unlike the thermal conductivity, the optimization of electrical conductivity may be attributed to doping effect, ordering on microstructure, and electron-electron interactions (*20*), further study is underway to elucidate the origin of the increase.

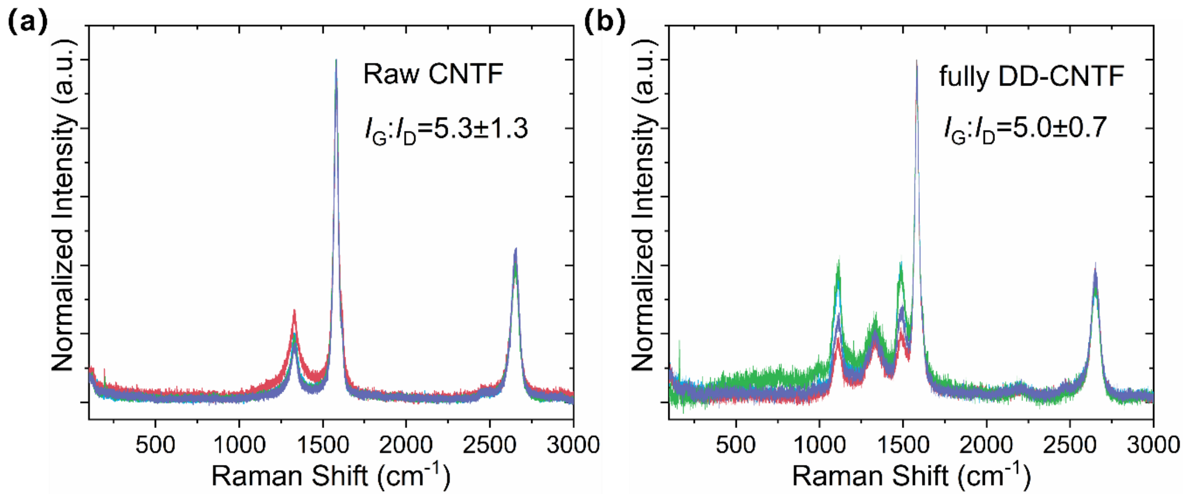

Fig. S7. Raman spectra of (a) raw CNTF and (b) fully DD-CNTF. The high performance fully DD-CNTF Although the raw CNTFs are only medium-grade crystalline materials (I_G:I_D =5.3±1.3), they can still be processed to produce high performance DD-CNTFs. Moreover, as the I_G:I_D for fully DD-CNTFs is 5.0±0.7, we have not observed obvious increase of defects after the Double-Drawing process. For the DD-CNTFs, extra peaks with positions ~1108 cm^{-1}, ~1487 cm^{-1} (and ~2186 cm^{-1} with low intensity) always appear. We have confirmed that the intensities of extra peaks are not related to the draw-ratio or the strength of the CNTFs. The intensities of extra peaks are always much lower than the D band. The highest intensity are shown here. We also confirmed that these peaks are not from CSA or chloroform itself. According to the EDX results of the CNTFs' surface (Fig. S12), we only find sulfur and chlorine as the extra elements appear after processing, which should originate from the impurities caused by the CSA to the fibers. We also find that by using multi-recycled chloroform as the processing liquid, the intensities of extra peaks seem to increase. Therefore, we suspect the extra peaks are from the tiny amount of impurity materials from the analytically pure chloroform which precipitated from chloroform when CSA dissolved in, and finally be left on the CNTFs with huge surface area. More dedicated experiments are underway to verify this conclusion.

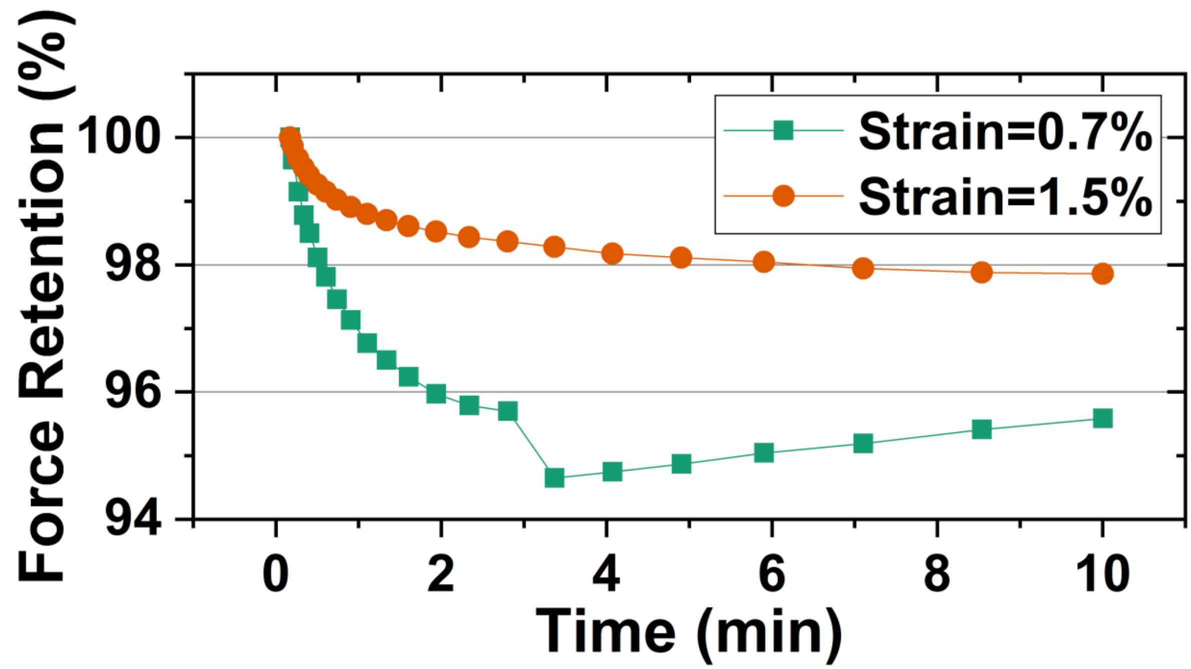

Fig. S8. Force retention of raw CNTF under tension. The strain of 0.7% and 1.5% is exerted onto raw CNTF to monitor the change of force. As shown, the force can be maintained to at least 95% within 10 mins.

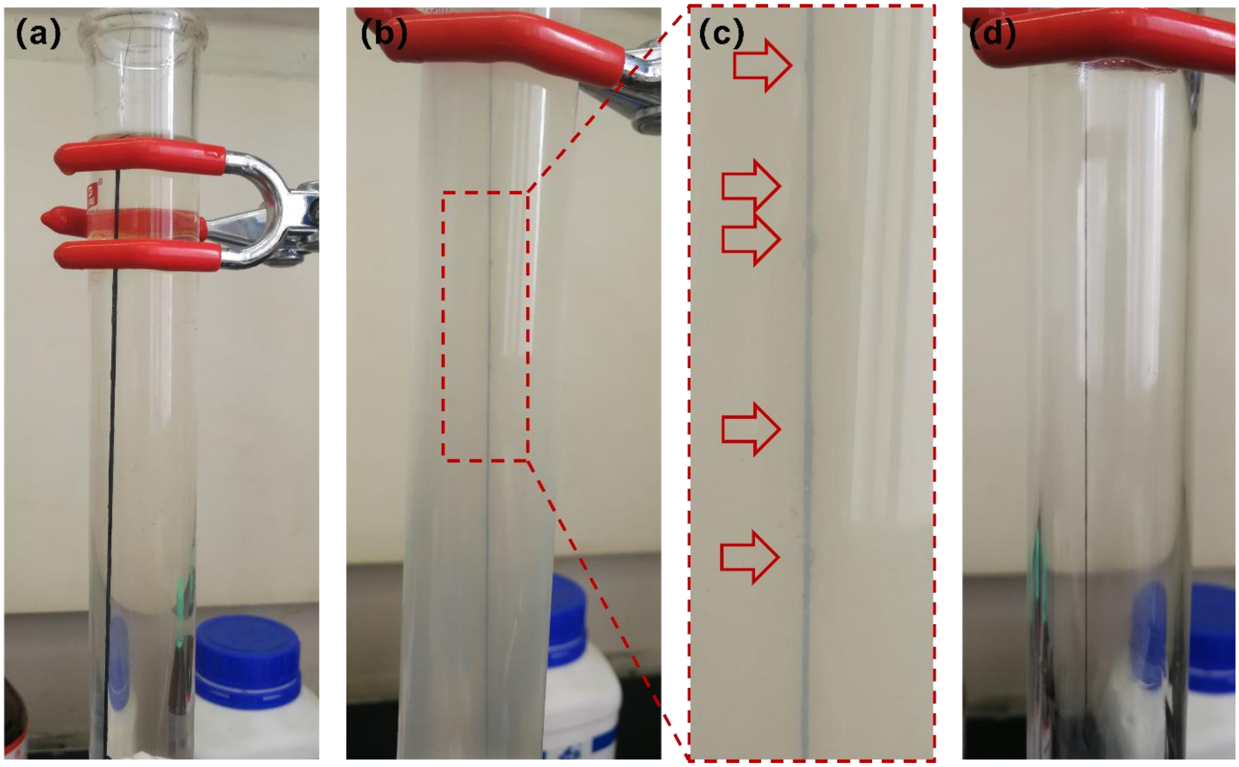

Figure S9. Photos of a CNTF in the dropping funnel filled with various solutions. (a) when immersed in CSA, the CNTF swells, (b) After the immersing fluid is exchanged from CSA to chloroform, pimples of CSA always appear on the surface of the CNTF (c), this is because the closely compacted tubes on the outer fiber layer hinders the diffusion of the remaining CSA from the inner layers. (d) As shown in air, after the Poisson Tightening, the fiber becomes uniform in diameter along the length.

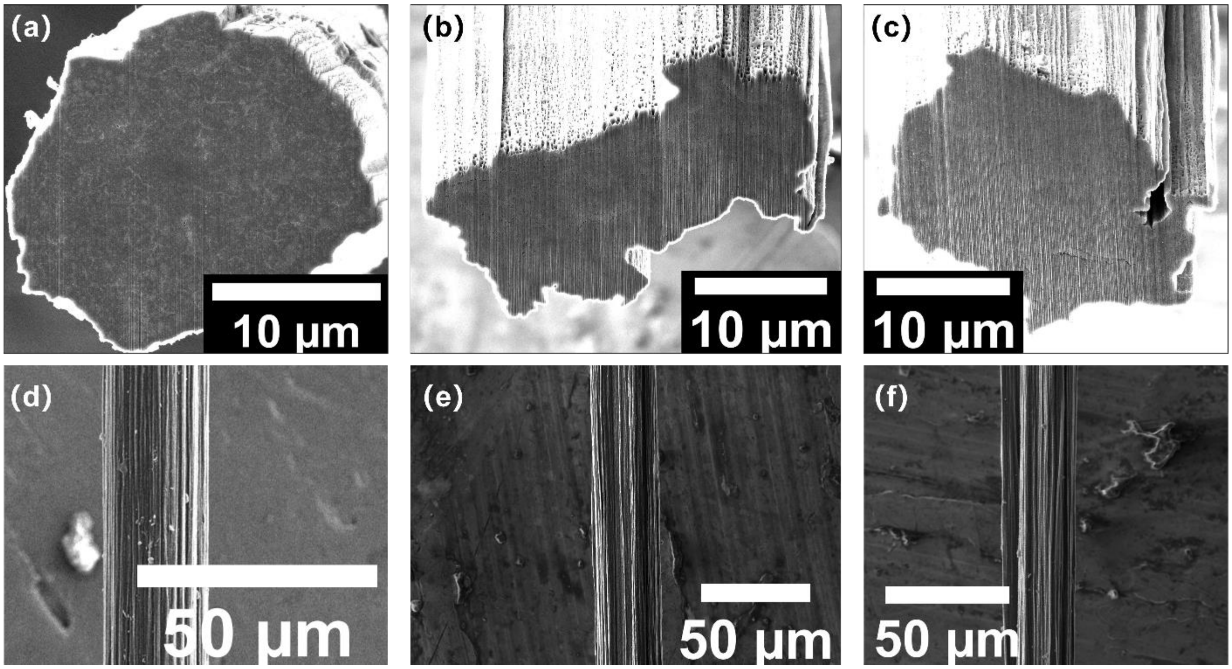

Figure S10. The comparison of cross-section and side surface between the fully Double-Drawn CNTF and two fully Single-Drawn CNTFs. After cut and finely polished by FIB, the cross-sections of (a) the fully DD-CNTF is much rounder than those of (b-c) two fully SD-CNTFs. Moreover, there are fewer wrinkles around the perimeter for (d) the fully DD-CNTF than those of (e-f) two fully SD-CNTFs. We think this is due to the radial force from the Poisson Tightening, which expels residual CSA as well as reshaping the fiber geometry.

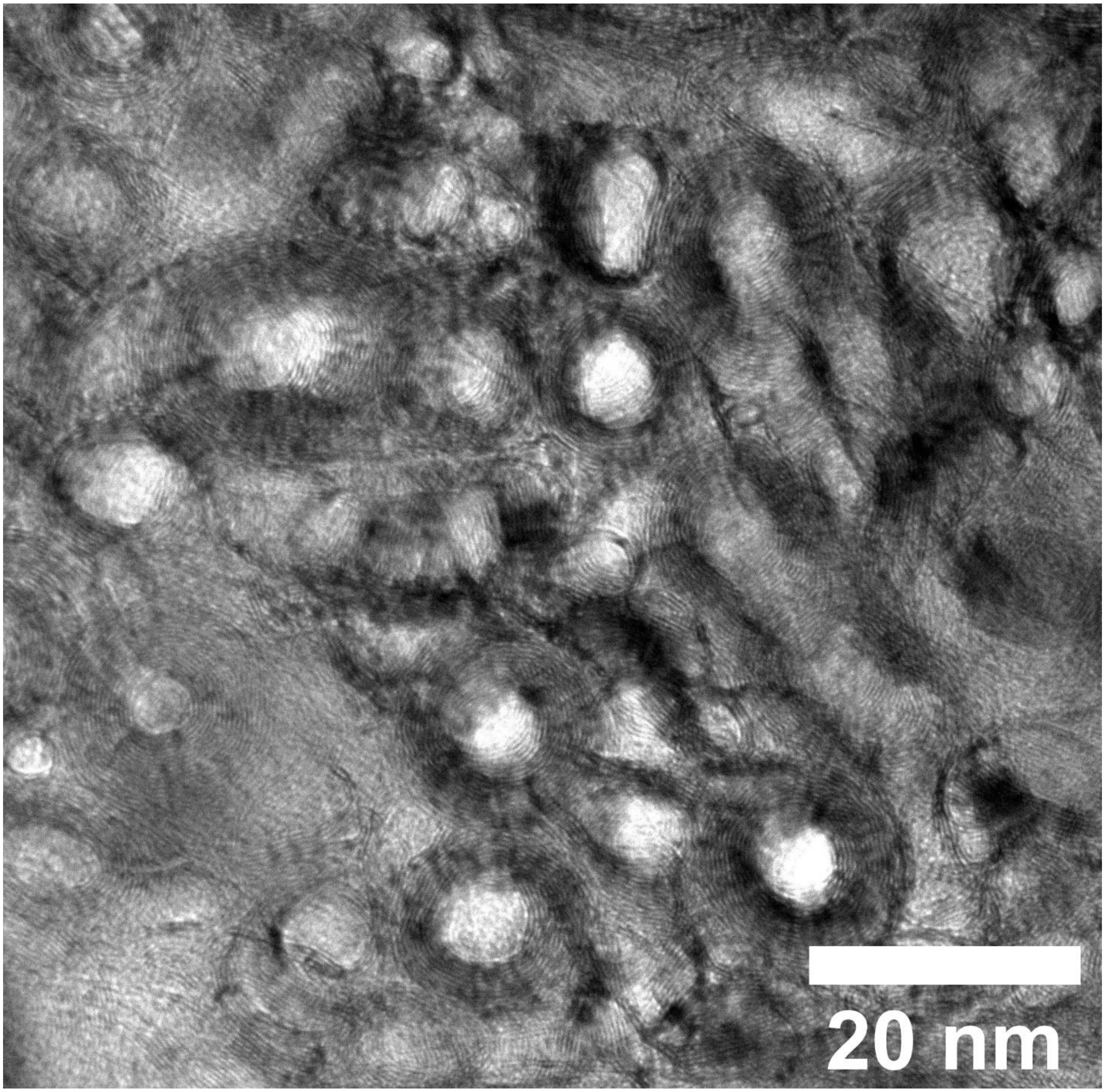

Figure S11. HRTEM image of the cross-section of iCNTs within raw CNTFs. iCNTs are multi-walled CNTs with outer diameter in the range of 11-15 nm, inner diameter in the range of 4-6 nm, and number of walls in the range of 7-15. Based on reported calculations (*32*), the densities of these multi-walled CNTs are in the range of 1.6-1.9 g cm^{-3}, with an average of 1.7 g cm^{-3}.

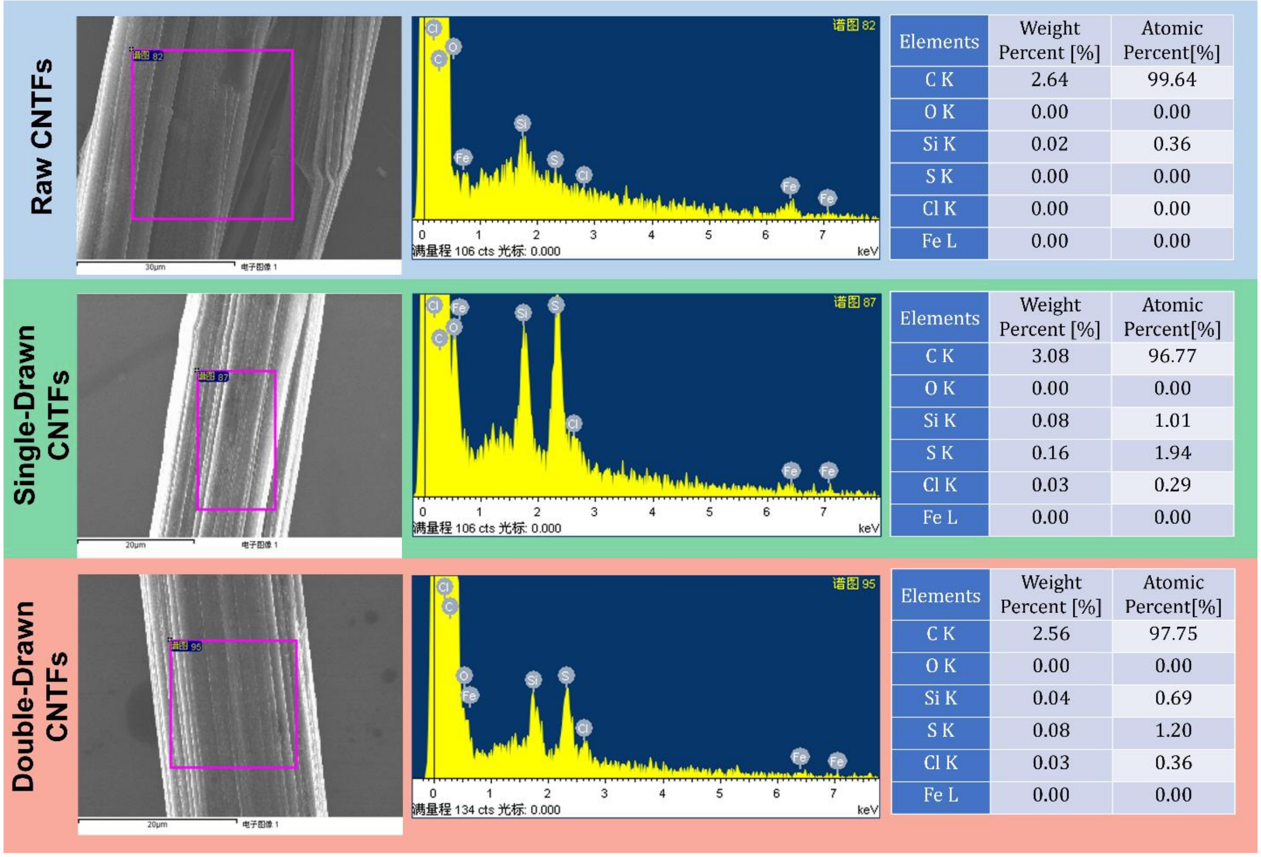

Raw CNTFs

Elements	Weight Percent [%]	Atomic Percent[%]
C K	2.64	99.64
O K	0.00	0.00
Si K	0.02	0.36
S K	0.00	0.00
Cl K	0.00	0.00
Fe L	0.00	0.00

Single-Drawn CNTFs

Elements	Weight Percent [%]	Atomic Percent[%]
C K	3.08	96.77
O K	0.00	0.00
Si K	0.08	1.01
S K	0.16	1.94
Cl K	0.03	0.29
Fe L	0.00	0.00

Double-Drawn CNTFs

Elements	Weight Percent [%]	Atomic Percent[%]
C K	2.56	97.75
O K	0.00	0.00
Si K	0.04	0.69
S K	0.08	1.20
Cl K	0.03	0.36
Fe L	0.00	0.00

Figure S12. EDX analysis of the raw, SD-, and DD-CNTFs. S and Cl signals detected on both SD- and DD-CNTFs originate from the CSA effects on the CNTFs. Si signal comes from the substrate underneath. There are no other elements observed on the processed CNTFs.

Science Advances

AAAS

Table S1. Summary of mechanical, thermal, and electrical properties of pure CNT fibers in our and others' work, and some commercial high-performance fibers. The data list is also used for the Ashby plot of main text.

Name [a)]	Comments		Density [g cm^{-3}]	Tenacity [N tex^{-1}]	Initial Modulus [N tex^{-1}]	Elongation [%]	Work of rupture [J g^{-1}]	Electrical Conductivity [MS m^{-1}]	Thermal Conductivity [W m^{-1} K^{-1}]
This Work-Fully DD	Fully Double-Drawn CNTF		1.40±0.01	3.22±0.07	138±6	3.9±0.2	75±5	1.07	354
This Work-Raw	Raw Direct-spun CNTF		0.66±0.15	1.23±0.08	53±5	5.0±0.5	43±6	0.16	106
SS-2017(*58*)	Solution-spun CNTF		1.50±0.10	1.54±0.09	160±40	1.8±0.2	17	8.50	-
SS-2004(*59*)	Solution-spun CNTF		1.11±0.07	0.10±0.01	108±9	-	-	-	21
SS-2013(*20, 58*)	Solution-spun CNTF with CCNI DWCNT		1.30±0.10	0.97	92±38	1.4±0.5	-	2.90	380±15
SS-2021(*36*)	Solution-spun CNTF		1.93±0.16	2.10±0.30	-	3.5±0.6	50±14	10.90±1.20	391±63
H-SS-2022(*39*)	Heat Solution-Spun CNTF	S·DW:1400℃	1.88	2.74±0.18	252±12	1.82±0.16	29.08±4.94	1.86±0.56	-
		S·DW:1700℃	1.71	3.84±0.42	368±13	1.53±0.21	33.76±7.09	2.22±0.34	482±63
		S·DW:2000℃	1.58	2.93±0.33	372±5	1.15±0.26	17.55±5.18	1.22±0.32	-
		S·DW:2400℃	1.43	2.22±0.20	420±2	0.50±0.17	5.59±1.03	0.33±0.21	-
		S·DW:2700℃	1.89	1.53±0.26	445±9.5	0.36±0.03	3.18±046	0.38±0.19	490±31.67
		SW:2700℃	1.80	0.34±0.07	184±11	0.18±0.07	-	0.22±0.18	305±5.23
		DW:2700℃	1.96	1.24±0.11	535±54	0.33±0.01	-	0.33±0.20	496±5.82
H-SS-2022-RAW(*39*)	Raw Solution-Spun CNTF	S·DWNT-Raw	1.92	2.10±0.17	155±9	2.55±0.37	39.38±6.69	10.88±0.77	380±21.46
		SWNT-Raw	1.6	0.62±0.06	137±30	1.31±0.18	-	3.52±0.48	213±42.87
		DWNT-Raw	2.10	1.10±0.16	83±8	2.04±0.38	-	8.19±0.42	406±8.69
D-SS-2022(*60*)	Densified Solution-Spun CNTF		1.97	2.55±0.2	-	-	-	11.20	398±27
GO-SS-2022(*40*)	GO interface hybrid Solution-Spun CNTF	GO: 0%	1.93±0.02	2.05	154	3.0	38.4±6.2	10.4±0.88	-
		GO: 5%	1.98±0.03	2.66	190	3.3	57.9±5.8	9.41±0.81	-
		GO: 10%	2.01±0.02	3.01	210	3.6	76.8±6.3	8.43±0.95	-
		GO: 20%	1.99±0.03	2.41	193	3.2	51.8±6.9	7.45±0.91	-
DS-IMDEA-2021(*61*)	Direct-spun CNTF with optimization synthesis parameters		-	2.10±0.13	107±7	5.25±0.55	63±9	0.338±0.053	-
DS-IMDEA-DW(*13*)	Direct-spun CNTF of collapsed DWCNT		-	1.70±0.30	61±7	-	100±30	-	-
DS-IMDEA-MW(*13*)	Direct-spun CNTF of few-layer MWCNT		-	1.10±0.20	64±16	-	80±40	-	-
DS-PUST-CSA(*62*)	CSA 13% stretched Direct-spun CNTF		-	2.19±0.25	176±22	3.3	39	-	-
DS-PUST-Raw(*62*)	Raw Direct-spun CNTF		-	0.95±0.13	76±8	5.5	50	-	-
DS-CAM-Den(*14*)	Acetone Direct-spun CNTF		-	1.10	50	3.8	13	-	-
DS-CAM-DogBone(*50*)	Dog Bone Direct-spun CNTF		-	2.20	160	-	46	-	-
VA-UT2004(*19*)	Twisted VA-CNTF		0.80	0.58	--	7.2	14	0.03	-
VA-UT2007(*63*)	Twisted VA-CNTF		0.80	0.88	--	6.0	27	-	-
VA-MIT(*37*)	Densified VA-CNTF		0.80	1.80	89	-	94	-	-
Toray T300(*1*)	PAN-based CF		1.76	2.01	131	1.5	16	0.06	10.5
Toray T700SC	PAN-based CF		1.80	2.72	128	2.1	-	0.06	9.6
Toray T800SC	PAN-based CF		1.80	3.27	163	2.0	-	0.08	11.3

Toray T1000GB	PAN-based CF	1.80	3.54	163	2.2	-	0.07	10.5
Toray T1100GC(*1*)	PAN-based CF	1.79	3.91	181	2.0	39	0.07	13
Toray M35JB(*1*)	PAN-based CF	1.75	2.58	196	1.3	18	0.09	38.9
Toray M40JB	PAN-based CF	1.77	2.49	213	1.2	-	0.10	66.9
Toray M60JB(*1*)	PAN-based CF	1.93	1.98	305	0.7	7	0.14	150.5
HexTow AS4(*1, 64*)	PAN-based CF	1.79	2.48	129	1.7	23	0.06	6.83
HexTow IM7	PAN-based CF	1.78	3.20	155	1.8	-	0.07	5.4
HexTow IM10	PAN-based CF	1.79	3.81	175	2.0	-	0.08	6.14
HexTow HM63	PAN-based CF	1.83	2.64	237	1.0	-	0.11	55
GRANOC YSH50A	Pitch-based CF	2.10	1.82	248	0.7	-	0.14	120
GRANOC YSH70A	Pitch-based CF	2.15	1.69	335	0.5	-	0.20	250
GRANOC YS80A	Pitch-based CF	2.17	1.67	362	0.5	-	0.20	320
GRANOC YS90A	Pitch-based CF	2.18	1.62	404	0.3	-	0.33	500
GRANOC YS95A	Pitch-based CF	2.19	1.61	420	0.3	-	0.45	600
DIALEAD K1352U	Pitch-based CF	2.12	1.70	292	0.6	-	0.15	140
DIALEAD K1392U	Pitch-based CF	2.15	1.72	353	0.5	-	0.20	210
DIALEAD K13C6U	Pitch-based CF	2.18	1.65	413	0.4	-	0.40	580
DIALEAD K13D2U	Pitch-based CF	2.20	1.68	425	0.4	-	0.67	800
Zylon AS(*64*)	PBO fiber	1.54	3.70	117	3.5	65	-	19
Zylon HM(*64*)	PBO fiber	1.56	3.70	172	2.5	46	-	23
Dyneema SK60	UHMWPE fiber	0.97	3.10	107	3.7	-	-	20
Dyneema SK75(*1, 64*)	UHMWPE fiber	0.97	3.74	133	3.5	63	-	14
Dyneema SK99	UHMWPE fiber	0.98	4.30	159	3.5	-	-	20
Vectran UM(*1*)	Polyarylate fiber	1.41	2.03	73	2.7	30	-	-
Vectran HT(*1*)	Polyarylate fiber	1.41	2.29	53	3.8	43	-	1.5
Kevlar 29	Polyamide fiber	1.44	2.03	49	3.6	36	-	0.04
Kevlar 49	Polyamide fiber	1.44	2.08	78	2.4	25	-	0.04
Kevlar 149(*24*)	Polyamide fiber	1.47	2.35	122	2.5	-	-	3
Technora(*1, 64*)	Polyamide fiber	1.39	2.40	54	4.2	42	-	-
C-Glass	Glass fiber	2.54	1.30	27	4.8	1.1	-	-
E-Glass(*1*)	Glass fiber	2.57	1.41	28	4.8	1.3	-	-
S-Glass(*1*)	Glass fiber	2.48	1.85	35	5.4	1.45	-	31
R-Glass	Glass fiber	2.54	1.63	34	4.8	-	-	37
Gold(*20*)	Metal	19.32	0.01	4	-	-	45.30	315
Silver(*20*)	Metal	10.50	0.01	7	-	-	62.00	427
Copper(*20*)	Metal	8.92	0.04	13	-	-	59.40	398
Nickel(*20*)	Metal	8.73	0.04	19	-	-	15.36	91.4
Aluminium(*20*)	Metal	2.72	0.04	25	-	-	47.60	238
Stainless steel	Metal	7.80	0.35	29	1.4	-	1.50	17

a) Most data of commercial fibers are based on the corresponding technical data from their company's websites or www.matweb.com

Table S2. Comparison of results from some related and recent works.

No.	Group	Publication	Comments	I_G:I_D	Raw fiber Tenacity [N/tex]	Enhanced Tenacity [a] [N/tex]	Linear Density [tex]		Linear Density Measurement Methods [a]	Toughness [J/g]	Thermal conductivity [W/(K m)]
							Before	After			
1	This Work	-	Double-Drawing the Direct-Spinning CNTF	~5	1.23±0.08	3.22±0.07	0.55±0.03	0.42±0.03	Direct Single-fiber Weighing	75±5	354
2	KIST (S. Korean) Hyeon Su Jeong & Seung Min Kim	*Nat. Comm.* (2019), 10, 2962	Single-Drawing the Direct-Spinning CNTF	~5	2.20±0.14	2.75±0.26	0.044	“changed negligibly” after even stretching 10%	Vibroscopic method without “stiffness correction” mentioned	No data	No data
				~12	2.12±0.19	3.88±0.28					
				~17	2.10±0.14	4.08±0.25					
3	KIST (S. Korean) Seung Min Kim	*Carbon* (2021), 172, 733-741	Single-Drawing the Direct-Spinning CNTF	No data	0.19	1.52	~6	~3	Direct Single-fiber Weighing	No data	No data
4	LG Chem R&D (S. Korean) Won Jae Lee	*ACS Appl. Mater. Inter.* (2020), 12, 13107−13115	Single-Drawing the Direct-Spinning CNTF	~13	2.2±0.10	5.5±0.84	0.19±0.02	0.13±0.02	Vibroscopic method without “stiffness correction” mentioned	No data	No data
5	IMDEA (Spain) Juan J. Vilatela	*Carbon* (2021), 179, 417-424	Direct-spinning CNTF with optimization synthesis parameters	~7	2.10±0.13	Not Applicable	0.05	Not Applicable	Vibroscopic method without “stiffness correction” mentioned	63±9	No data
6	KIST (S. Korean) Bon-Cheol Ku	*Sci. Adv.* (2022), 8, eabn0939	Heating the liquid crystal Solution-Spinning CNTF: S·DW:1700°C	~50	2.10±0.17	3.84±0.42	0.25±0.02	0.19±0.02	Vibroscopic method without “stiffness correction” mentioned	33.76±7.09	482±63
			S·DW:2000°C			2.93±0.33	0.25±0.02	0.20±0.02		17.55±5.18	No data
			S·DW:2700°C			1.53±0.26	0.25±0.02	0.19±0.01		3.18±046	490±32
			SW:2700°C		0.62±0.06	0.34±0.07	0.65±0.01	0.54±0.01		No data	305±5
			DW:2700°C		1.10±0.16	1.24±0.11	0.49±0.02	0.37±0.02		No data	496±6
7	KIST (S. Korean) Bon-Cheol Ku	*Carbon* (2022), 196, 59–69	Densified the liquid crystal Solution-Spinning CNTF	~50	0.51	2.55±0.2	No data	~0.3	Vibroscopic method without “stiffness correction” mentioned	No data	398±27
8	Rice (USA) Matteo Pasquali	*Carbon* (2021), 171, 689-694	Improved liquid crystal Solution-Spinning CNTF	>54	Not Applicable	2.1±0.3	Not Applicable	0.12±0.01	Vibroscopic method without “stiffness correction”	50±14	390±60
9	Rice (USA) Matteo Pasquali	*Sci. Adv.* (2022), 8, eabm3285	liquid crystal Solution-Spinning CNTF from friendly acid	~40	Not Applicable	0.78	Not Applicable	Not Applicable	Vibroscopic method without “stiffness correction” mentioned	No data	No data

10	KIST (S. Korean) Bon-Cheol Ku	*Adv. Sci.* (2022), 2203008	GO interface hybrid Solution-Spinning CNTF	GO: 5 vol%	43	1.93±0.02	1.98±0.03	No data	No data	Vibroscopic method without "stiffness correction" mentioned	57.9±5.8	No data
				GO: 10 vol%			2.01±0.02		No data		76.8±6.3	No data
				GO: 20 vol%			1.99±0.03		No data		51.8±6.9	No data

[a] As mentioned in S2, ASTM D1577−07(2018) OPTION C, the standard behind the Vibroscopic methods to measure the linear density, "stiffness correction" needed to be done, and the stiffness factor $\alpha \equiv (4EI/l^2F_{PT})^{1/2}$, is used to estimate the deviation of CNTFs from a flexible string. Based on the reported data comes from the corresponding articles, results being >5% overestimated are in red font.

REFERENCES AND NOTES

1. A. Mikhalchan, J. J. Vilatela, A perspective on high-performance CNT fibres for structural composites. *Carbon* **150**, 191–215 (2019).

2. M. F. Yu, O. Lourie, M. J. Dyer, K. Moloni, T. F. Kelly, R. S. Ruoff, Strength and breaking mechanism of multiwalled carbon nanotubes under tensile load. *Science* **287**, 637–640 (2000).

3. B. Peng, M. Locascio, P. Zapol, S. Li, S. L. Mielke, G. C. Schatz, H. D. Espinosa, Measurements of near-ultimate strength for multiwalled carbon nanotubes and irradiation-induced crosslinking improvements. *Nat. Nanotechnol.* **3**, 626–631 (2008).

4. X. Zhang, W. Lu, G. Zhou, Q. Li, Understanding the mechanical and conductive properties of carbon nanotube fibers for smart electronics. *Adv. Mater.* **32**, 1902028 (2020).

5. P. Kim, L. Shi, A. Majumdar, P. L. McEuen, Thermal transport measurements of individual multiwalled nanotubes. *Phys. Rev. Lett.* **87**, 215502 (2001).

6. N. Komatsu, Y. Ichinose, O. S. Dewey, L. W. Taylor, M. A. Trafford, Y. Yomogida, G. Wehmeyer, M. Pasquali, K. Yanagi, J. Kono, Macroscopic weavable fibers of carbon nanotubes with giant thermoelectric power factor. *Nat. Commun.* **12**, 4931 (2021).

7. M. F. L. L. De Volder, S. H. Tawfick, R. H. Baughman, A. J. Hart, Carbon nanotubes: Present and future commercial applications. *Science* **339**, 535–539 (2013).

8. J. J. Vilatela, A. H. Windle, Yarn-like carbon nanotube fibers. *Adv. Mater.* **22**, 4959–4963 (2010).

9. B. Alemán, V. Reguero, B. Mas, J. J. Vilatela, Strong carbon nanotube fibers by drawing inspiration from polymer fiber spinning. *ACS Nano* **9**, 7392–7398 (2015).

10. Huntsman, An Aggressive Miralon Scale Up Program, Carbon Hub, Rice University (2022); https://www.huntsman.com/products/detail/344/miralon/dispersed-products.

11. Y. Bai, R. Zhang, X. Ye, Z. Zhu, H. Xie, B. Shen, D. Cai, B. Liu, C. Zhang, Z. Jia, S. Zhang, X. Li, F. Wei, Carbon nanotube bundles with tensile strength over 80 GPa. *Nat. Nanotechnol.* **13**, 589–595 (2018).

12. M.-F. Yu, B. S. Files, S. Arepalli, R. S. Ruoff, Tensile loading of ropes of single wall carbon nanotubes and their mechanical properties. *Phys. Rev. Lett.* **84**, 5552–5555 (2000).

13. J. C. Fernández-Toribio, B. Alemán, Á. Ridruejo, J. J. Vilatela, Tensile properties of carbon nanotube fibres described by the fibrillar crystallite model. *Carbon* **133**, 44–52 (2018).

14. K. Koziol, J. Vilatela, A. Moisala, M. Motta, P. Cunniff, M. Sennett, A. Windle, High-performance carbon nanotube fiber. *Science* **318**, 1892–1895 (2007).

15. J. N. Wang, X. G. Luo, T. Wu, Y. Chen, High-strength carbon nanotube fibre-like ribbon with high ductility and high electrical conductivity. *Nat. Commun.* **5**, 3848 (2014).

16. W. Xu, Y. Chen, H. Zhan, J. N. Wang, High-strength carbon nanotube film from improving alignment and densification. *Nano Lett.* **16**, 946–952 (2016).

17. T. Q. Tran, Z. Fan, P. Liu, S. M. Myint, H. M. Duong, Super-strong and highly conductive carbon nanotube ribbons from post-treatment methods. *Carbon* **99**, 407–415 (2016).

18. R. J. Headrick, D. E. Tsentalovich, J. Berdegué, E. A. Bengio, L. Liberman, O. Kleinerman, M. S. Lucas, Y. Talmon, M. Pasquali, Structure–property relations in carbon nanotube fibers by downscaling solution processing. *Adv. Mater.* **30**, 1–8 (2018).

19. M. Zhang, K. R. Atkinson, R. H. Baughman, Multifunctional carbon nanotube yarns by downsizing an ancient technology. *Science* **306**, 1358–1361 (2004).

20. N. Behabtu, C. C. Young, D. E. Tsentalovich, O. Kleinerman, X. Wang, A. W. K. Ma, E. A. Bengio, R. F. Ter Waarbeek, J. J. De Jong, R. E. Hoogerwerf, S. B. Fairchild, J. B. Ferguson, B. Maruyama, J. Kono, Y. Talmon, Y. Cohen, M. J. Otto, M. Pasquali, Strong, light, multifunctional fibers of carbon nanotubes with ultrahigh conductivity. *Science* **339**, 182–186 (2013).

21. J. Lee, D. M. Lee, Y. Jung, J. Park, H. S. Lee, Y. K. Kim, C. R. Park, H. S. Jeong, S. M. Kim, Direct spinning and densification method for high-performance carbon nanotube fibers. *Nat. Commun.* **10**, 1–10 (2019).

22. V. A. Davis, A. N. G. Parra-Vasquez, M. J. Green, P. K. Rai, N. Behabtu, V. Prieto, R. D. Booker, J. Schmidt, E. Kesselman, W. Zhou, H. Fan, W. W. Adams, R. H. Hauge, J. E. Fischer, Y. Cohen, Y. Talmon, R. E. Smalley, M. Pasquali, True solutions of single-walled carbon nanotubes for assembly into macroscopic materials. *Nat. Nanotechnol.* **4**, 830–834 (2009).

23. A. N. G. Parra-Vasquez, N. Behabtu, M. J. Green, C. L. Pint, C. C. Young, J. Schmidt, E. Kesselman, A. Goyal, P. M. Ajayan, Y. Cohen, Y. Talmon, R. H. Hauge, M. Pasquali, Spontaneous dissolution of ultralong single-and multiwalled carbon nanotubes. *ACS Nano* **4**, 3969–3978 (2010).

24. J. W. S. Hearle, W. E. Morton, *Physical Properties of Textile Fibres* (Elsevier, 2008).

25. D.-M. Lee, J. Park, J. Lee, S.-H. Lee, S.-H. Kim, S. M. Kim, H. S. Jeong, Improving mechanical and physical properties of ultra-thick carbon nanotube fiber by fast swelling and stretching process. *Carbon* **172**, 733–741 (2021).

26. S. Ramesh, L. M. Ericson, V. A. Davis, R. K. Saini, C. Kittrell, M. Pasquali, W. E. Billups, W. W. Adams, R. H. Hauge, R. E. Smalley, Dissolution of pristine single walled carbon nanotubes in superacids by direct protonation. *J. Phys. Chem. B* **108**, 8794–8798 (2004).

27. N. Kateris, P. Kloza, R. Qiao, J. A. Elliott, A. M. Boies, From collisions to bundles: An adaptive coarse-grained model for the aggregation of high-aspect-ratio carbon nanotubes. *J. Phys. Chem. C* **124**, 8359–8370 (2020).

28. A. M. Boies, C. Hoecker, A. Bhalerao, N. Kateris, J. de La Verpilliere, B. Graves, F. Smail, Agglomeration dynamics of 1D materials: Gas-phase collision rates of nanotubes and nanorods. *Small* **15**, e1900520 (2019).

29. B. I. Yakobson, L. S. Couchman, Persistence length and nanomechanics of random bundles of nanotubes. *J. Nanopart. Res.* **8**, 105–110 (2006).

30. J. C. Stallard, W. Tan, F. R. Smail, T. S. Gspann, A. M. Boies, N. A. Fleck, The mechanical and electrical properties of direct-spun carbon nanotube mats. *Extrem. Mech. Lett.* **21**, 65–75 (2018).

31. J. Park, J. Lee, D.-M. Lee, S.-H. Lee, H. S. Jeong, K.-H. Lee, S. M. Kim, Mathematical model for the dynamic mechanical behavior of carbon nanotube yarn in analogy with hierarchically structured bio-materials. *Carbon* **152**, 151–158 (2019).

32. C. Laurent, E. Flahaut, A. Peigney, The weight and density of carbon nanotubes versus the number of walls and diameter. *Carbon* **48**, 2994–2996 (2010).

33. N. Fakhri, D. A. Tsyboulski, L. Cognet, R. Bruce Weisman, M. Pasquali, Diameter-dependent bending dynamics of single-walled carbon nanotubes in liquids. *Proc. Natl. Acad. Sci. U.S.A.* **106**, 14219–14223 (2009).

34. R. S. Prasher, X. J. Hu, Y. Chalopin, N. Mingo, K. Lofgreen, S. Volz, F. Cleri, P. Keblinski, Turning carbon nanotubes from exceptional heat conductors into insulators. *Phys. Rev. Lett.* **102**, 105901 (2009).

35. M. S. Dresselhaus, P. C. Eklund, Phonons in carbon nanotubes. *Adv. Phys.* **49**, 705–814 (2000).

36. L. W. Taylor, O. S. Dewey, R. J. Headrick, N. Komatsu, N. M. Peraca, G. Wehmeyer, J. Kono, M. Pasquali, Improved properties, increased production, and the path to broad adoption of carbon nanotube fibers. *Carbon* **171**, 689–694 (2021).

37. F. A. Hill, T. F. Havel, A. J. Hart, C. Livermore, Enhancing the tensile properties of continuous millimeter-scale carbon nanotube fibers by densification. *ACS Appl. Mater. Interfaces* **5**, 7198–7207 (2013).

38. R. J. Headrick, S. M. Williams, C. E. Owens, L. W. Taylor, O. S. Dewey, C. J. Ginestra, L. Liberman, A. M. Ya'akobi, Y. Talmon, B. Maruyama, G. H. McKinley, A. J. Hart, M. Pasquali, Versatile acid solvents for pristine carbon nanotube assembly. *Sci. Adv.* **8**, eabm3285 (2022).

39. D. Lee, S. G. Kim, S. Hong, C. Madrona, Y. Oh, M. Park, N. Komatsu, L. W. Taylor, B. Chung, J. Kim, J. Y. Hwang, J. Yu, D. S. Lee, H. S. Jeong, N. H. You, N. D. Kim, D.-Y. Kim, H. S. Lee, K.-H. Lee, J. Kono, G. Wehmeyer, M. Pasquali, J. J. Vilatela, S. Ryu, B.-C. Ku, Ultrahigh strength, modulus, and conductivity of graphitic fibers by macromolecular coalescence. *Sci. Adv.* **8**, eabn0939 (2022).

40. S. G. Kim, S. J. Heo, J.-G. Kim, S. Kim, D. Lee, M. Kim, N. D. Kim, D.-Y. Kim, J. Y. Hwang, H. G. Chae, B.-C. Ku, Ultrastrong hybrid fibers with tunable macromolecular interfaces of graphene oxide and carbon nanotube for multifunctional applications. *Adv. Sci.* **9**, 2203008 (2022).

41. C.-C. Chang, I.-K. Hsu, M. Aykol, W.-H. Hung, C.-C. Chen, S. B. Cronin, A new lower limit for the ultimate breaking strain of carbon nanotubes. *ACS Nano* **4**, 5095–5100 (2010).

42. S. B. Cronin, A. K. Swan, M. S. Ünlü, B. B. Goldberg, M. S. Dresselhaus, M. Tinkham, Measuring the uniaxial strain of individual single-wall carbon nanotubes: Resonance raman spectra of atomic-force-microscope modified single-wall nanotubes. *Phys. Rev. Lett.* **93**, 167401 (2004).

43. R. Saito, M. Hofmann, G. Dresselhaus, A. Jorio, M. S. Dresselhaus, Raman spectroscopy of graphene and carbon nanotubes. *Adv. Phys.* **60**, 413–550 (2011).

44. X. Zhang, L. Song, L. Cai, X. Tian, Q. Zhang, X. Qi, W. Zhou, N. Zhang, F. Yang, Q. Fan, Y. Wang, H. Liu, X. Bai, W. Zhou, S. Xie, Optical visualization and polarized light absorption of the single-wall carbon nanotube to verify intrinsic thermal applications. *Light Sci. Appl.* **4**, e318 (2015).

45. R. Kumar, S. B. Cronin, Raman scattering of carbon nanotube bundles under axial strain and strain-induced debundling. *Phys. Rev. B* **75**, 155421 (2007).

46. W. W. Adams, R. K. Eby, High-performance polymer fibers. *MRS Bull.* **12**, 22–26 (1987).

47. N. Gupta, J. M. Alred, E. S. Penev, B. I. Yakobson, Universal strength scaling in carbon nanotube bundles with frictional load transfer. *ACS Nano* **15**, 1342–1350 (2021).

48. Y. Chalopin, S. Volz, N. Mingo, Upper bound to the thermal conductivity of carbon nanotube pellets. *J. Appl. Phys.* **105**, 084301 (2009).

49. Y. L. Li, I. A. Kinloch, A. H. Windle, Direct spinning of carbon nanotube fibers from chemical vapor deposition synthesis. *Science* **304**, 276–278 (2004).

50. M. Motta, A. Moisala, I. A. Kinloch, A. H. Windle, High performance fibres from "dog bone" carbon nanotubes. *Adv. Mater.* **19**, 3721–3726 (2007).

51. X. Zhang, W. Tan, F. Smail, M. De Volder, N. Fleck, A. Boies, High-fidelity characterization on anisotropic thermal conductivity of carbon nanotube sheets and on their effects of thermal enhancement of nanocomposites. *Nanotechnology* **29**, 365708 (2018).

52. W. Zhou, Q. Fan, Q. Zhang, L. Cai, K. Li, X. Gu, F. Yang, N. Zhang, Y. Wang, H. Liu, W. Zhou, S. Xie, High-performance and compact-designed flexible thermoelectric modules enabled by a reticulate carbon nanotube architecture. *Nat. Commun.* **8**, 14886 (2017).

53. W. B. Zhou, Q. X. Fan, Q. Zhang, K. W. Li, L. Cai, X. G. Gu, F. Yang, N. Zhang, Z. J. Xiao, H. L. Chen, S. Q. Xiao, Y. C. Wang, H. P. Liu, W. Y. Zhou, S. S. Xie, Ultrahigh-power-factor carbon nanotubes and an ingenious strategy for thermoelectric performance evaluation. *Small* **12**, 3407–3414 (2016).

54. M. Damnjanović, I. Milošević, T. Vuković, R. Sredanović, Full symmetry, optical activity, and potentials of single-wall and multiwall nanotubes. *Phys. Rev. B* **60**, 2728–2739 (1999).

55. D. J. Montgomery, W. T. Milloway, The vibroscopic method for determination of fiber cross-sectional area. *Text. Res. J.* **22**, 729–735 (1952).

56. E. T. L. Voong, D. J. Montgomery, Experimental study of stiffness and nonuniformity in the vibroscopic determination of fiber cross-sectional area. *Text. Res. J.* **23**, 821–830 (1953).

57. T. H. Loeber, B. Laegel, S. Wolff, S. Schuff, F. Balle, T. Beck, D. Eifler, J. H. Fitschen, G. Steidl, Reducing curtaining effects in FIB/SEM applications by a goniometer stage and an image processing method. *J. Vac. Sci. Technol. B* **35**, 06GK01 (2017).

58. D. E. Tsentalovich, R. J. Headrick, F. Mirri, J. Hao, N. Behabtu, C. C. Young, M. Pasquali, Influence of carbon nanotube characteristics on macroscopic fiber properties. *ACS Appl. Mater. Interfaces* **9**, 36189–36198 (2017).

59. L. M. Ericson, H. Fan, H. Peng, V. A. Davis, W. Zhou, J. Sulpizio, Y. Wang, R. Booker, J. Vavro, C. Guthy, A. N. G. Parra-Vasquez, M. J. Kim, S. Ramesh, R. K. Saini, C. Kittrell, G. Lavin, H. Schmidt, W. W. Adams, W. E. Billups, M. Pasquali, W. Hwang, R. H. Hauge, J. E. Fischer, R. E. Smalley, Macroscopic, neat, single-walled carbon nanotube fibers. *Science* **305**, 1447–1450 (2004).

60. S. S. G. Kim, G. M. Choi, H. D. Jeong, D. Lee, S. S. G. Kim, K.-H. Ryu, S. Lee, J. Kim, J. Y. Hwang, N. D. Kim, D.-Y. Kim, H. S. Lee, B.-C. Ku, Hierarchical structure control in solution spinning for strong and multifunctional carbon nanotube fibers. *Carbon* **196**, 59–69 (2022).

61. A. Mikhalchan, M. Vila, L. Arévalo, J. J. Vilatela, Simultaneous improvements in conversion and properties of molecularly controlled CNT fibres. *Carbon* **179**, 417–424 (2021).

62. H. Cho, H. Lee, E. Oh, S.-H. H. Lee, J. Park, H. J. Park, S.-B. B. Yoon, C.-H. H. Lee, G.-H. H. Kwak, W. J. Lee, J. E. J. Kim, J. E. J. Kim, K.-H. H. Lee, Hierarchical structure of carbon nanotube fibers, and the change of structure during densification by wet stretching. *Carbon* **136**, 409–416 (2018).

63. K. R. Atkinson, S. C. Hawkins, C. Huynh, C. Skourtis, J. Dai, M. Zhang, S. Fang, A. A. Zakhidov, S. B. Lee, A. E. Aliev, C. D. Williams, R. H. Baughman, Multifunctional carbon nanotube yarns and transparent sheets: Fabrication, properties, and applications. *Phys. B Condens. Matter* **394**, 339–343 (2007).

64. X. Wang, V. Ho, R. A. Segalman, D. G. Cahill, Thermal conductivity of high-modulus polymer fibers. *Macromolecules* **46**, 4937–4943 (2013).
